# High-resolution tip-enhanced Raman scattering probes sub-molecular density changes

**DOI:** 10.1038/s41467-019-10618-x

**Published:** 2019-06-12

**Authors:** Xing Chen, Pengchong Liu, Zhongwei Hu, Lasse Jensen

**Affiliations:** Department of Chemistry, Pennsylvania State University, University Park, Pennsylvania 16802 USA

**Keywords:** Method development, Raman spectroscopy, Raman spectroscopy

## Abstract

Tip-enhanced Raman spectroscopy (TERS) exhibits new selection rule and sub-nanometer spatial resolution, which is attributed to the plasmonic near-field confinement. Despite recent advances in simulations of TERS spectra under highly confined fields, a simply physical mechanism has remained elusive. In this work we show that single-molecule TERS images can be explained by local sub-molecular density changes induced by the confined near-field during the Raman process. The local sub-molecular density changes determine the spatial resolution in TERS and the gradient-based selection rule. Using this approach we find that the four-fold symmetry of *meso*-tetrakis(3,5-di-*tert*-butylphenyl)porphyrin (H_2_TBPP) TERS images observed in experiments arises from the combination of degenerate normal modes localized in the functional side groups rather than the porphyrin ring as previously considered. As an illustration of the potential of the method, we demonstrate how this new theory can be applied to microscopic structure characterization.

## Abstract (web summary)

Despite recent advances in simulations of tip-enhanced Raman spectroscopy (TERS) under highly confined fields, a simply physical mechanism has remained elusive. Here, the authors show that single molecule TERS images can be explained by local sub-molecular density changes induced by the confined near-field during the Raman process.

## Introduction

Tip-enhanced Raman spectroscopy (TERS) is a powerful technique to measure molecular properties with microscopic precision^1–11^. TERS measurements provide much richer information than traditional Raman spectroscopy, which is enabled by using a sharp metallic tip to probe the molecules in a sub-nanometer junction^12–14^. The sharp metallic tip confines the plasmonic near field in an extremely small volume, where the field-gradient effects become prominent leading to the significantly modified selection rule in TERS^15^. TERS spectra vary with the spatial movement of the tip. Many works reported such spatial resolutions achieve nanometer and sub-nanometer scales when atomically sharp tips are used^16–23^. One prime demonstration of these unique features is high-resolution TERS imaging. A TERS image of a given normal mode is a 2-dimensional (2D) mapping of the TERS intensities varying with the position of the tip^24,25^. The TERS images of different normal modes are predicted to be different, which contain the information of the unique selection rules in TERS^26^. Therefore, rationalizing the mode specificity and spatial variation of TERS imaging is necessary for fully understanding the physical mechanism of high-resolution TERS.

Many theoretical modeling works were carried out to simulate TERS images by calculating the molecular responses to narrowly distributed near fields^24,27–29^. It was reported that atomic resolution can be achieved when the near-field confinement reaches a few Ångstroms in diameter, and each normal mode can be uniquely resolved^26,30,31^. TERS images in both simulations and experiments suggested strong correlations between the hotspots distributions and the vibrating atoms. However, the molecular response is a non-local property and thus not easily localized on individual atoms. Therefore, despite the success in simulating TERS images, existing theories do not provide clear and consistent explanations on how the vibrating atoms locally affect the TERS intensities.

In this work, we address the question of what local property of a molecule is probed by the TERS tip. We demonstrate that sub-molecular density changes are probed by the confined near field in TERS and lead to the varying Raman intensities over normal modes and space. The probed density changes, which we define as Raman polarizability density, is a truly local property and is strongly correlated with the given vibrational mode. The density distribution is extracted from a small volume defined by the highly confined near field, leading to the spatially variant TERS intensities. This approach offers a clear explanation for the mode specificity and spatial variation of TERS signals. We show that the proposed mechanism accurately reproduces atomistic simulations and experimental results, and more importantly provides intuitive interpretations of TERS images. In the end we demonstrate how TERS imaging combined with the new theory can be applied to microscopic characterization and its potential to compete with scanning tunneling microscopy (STM).

## Results

### Locally integrated Raman polarizability density

The method was adopted in this work, namely locally integrated Raman polarizability density (LIRPD). All the molecular properties and the near field are dependent on the frequency of the external field, and are in tensor format. The explicit notations of frequency and the tensor subscripts are omitted for simplicity. A detailed justification of the method is provided in Supplementary Methods.

The concept of distributed polarizability density was first introduced by Maaskant and Oosterhoff in the theory of optical rotation^32^, and was later generalized by Hunt^33,34^. Briefly, the molecular polarizability *α* can be expressed as a spatial integration of a polarizability density *ρ*^(*α*)^,

1 $$\alpha = - {\int} {\rho ^{(\alpha )}} ({\mathbf{r}})\cdot d{\mathbf{r}} = - {\int} {\hat \mu ^{{\mathrm{eff}}}} \cdot \delta \rho ({\mathbf{r}})\cdot d{\mathbf{r}},$$

where *δρ*(**r**) is the linear change in the electron density of a molecule due to an external electric field, $\hat \mu ^{{\mathrm{eff}}}$ is the effective dipole operator, and **r** is a vector in space. The polarizability density, *ρ*^(*α*)^(**r**), is a local property as it’s derived from the electron density distribution, which is different from the definition in refs. ^33,34^. However, the concept of “polarizability density” is similar as its spatial integral gives rise to the molecular polarizability.

In the linear-response time-dependent density functional theory, the induced electron density of the molecule due to an electric field perturbation is expressed as

2 $$\delta \rho ({\mathbf{r}}) = {\int} \chi ({\mathbf{r}},{\mathbf{r}}\prime )\hat v^{{\mathrm{pert}}}({\mathbf{r}}\prime )d{\mathbf{r}}\prime ,$$

where *χ*(**r**, **r**′) is the density-density linear response function^35^ and $\hat v^{{\mathrm{pert}}}({\mathbf{r}}\prime )$ is the perturbation operator. Because the confined near field dominates the field distribution in the TERS junction, we can represent both the perturbation and effective dipole operators as the product of the near field distribution **F**(**r** − **R**) centered at **R** and a free-molecule operator in the unit external field,

3 $$\begin{array}{l}\hat \mu ^{{\mathrm{eff}}}({\mathbf{r}}) = - {\mathbf{F}}({\mathbf{r}} - {\mathbf{R}})\cdot \hat \mu ,\\ \hat v^{{\mathrm{pert}}}({\mathbf{r}}\prime - {\mathbf{R}}) = - {\mathbf{F}}({\mathbf{r}}\prime - {\mathbf{R}})\cdot \hat \mu ({\mathbf{r}}\prime ).\end{array}$$

Here $\hat \mu$ is the dipole operator, and the perturbation operator entails the plasmon-induced near field.

Combining the first three equations, we obtain the molecular polarizability that is now dependent on the tip position,

4 $$\begin{array}{c}\alpha ({\mathbf{R}}) = {\int} {\hat \mu ^{{\mathrm{eff}}}} ({\mathbf{r}})[{\int} \chi ({\mathbf{r}},{\mathbf{r}}\prime )\hat v^{{\mathrm{pert}}}({\mathbf{r}}\prime )d{\mathbf{r}}\prime ]d{\mathbf{r}}\\ = {\int} {\mathbf{F}} ({\mathbf{r}} - {\mathbf{R}})\hat \mu [{\int} \chi ({\mathbf{r}},{\mathbf{r}}\prime ,\omega )\hat \mu ({\mathbf{r}}\prime ){\mathbf{F}}({\mathbf{r}}\prime - {\mathbf{R}})d{\mathbf{r}}\prime ]d{\mathbf{r}}.\end{array}$$

Because the near field is highly confined in high-resolution TERS, the induced density away from the near-field center diminishes quickly. Thus, we make a local approximation to the induced density perturbed by the near field, and take the near-field distribution out of the inner integral. Then we obtain the molecular polarizability in the form of locally integrated polarizability density,

5 $$\begin{array}{c}\alpha ({\mathbf{R}}) = {\int} {\mathbf{F}} ({\mathbf{r}} - {\mathbf{R}})\hat \mu [{\int} \chi ({\mathbf{r}},{\mathbf{r}}\prime ,\omega )\hat \mu ({\mathbf{r}}\prime ){\mathbf{F}}({\mathbf{r}}\prime - {\mathbf{R}})d{\mathbf{r}}\prime ]d{\mathbf{r}}\\ \mathop { \approx }\limits^{{\mathrm{local}}} {\int} {\mathbf{F}} ({\mathbf{r}} - {\mathbf{R}})\hat \mu [{\int} {\chi ^{{\mathrm{free}}}} ({\mathbf{r}},{\mathbf{r}}\prime ,\omega )\hat \mu ({\mathbf{r}}\prime )d{\mathbf{r}}\prime ]{\mathbf{F}}({\mathbf{r}} - {\mathbf{R}})d{\mathbf{r}}\\ = {\int} {{\mathbf{F}}({\mathbf{r}} - {\mathbf{R}})} \cdot \hat \mu {\mkern 1mu} \delta \rho ^{{\mathrm{free}}}({\mathbf{r}})\cdot {\mathbf{F}}({\mathbf{r}} - {\mathbf{R}})\cdot d{\mathbf{r}}\\ = {\int} {{\mathbf{F}}({\mathbf{r}} - {\mathbf{R}})} \cdot \rho ^{(\alpha )}({\mathbf{r}})\cdot {\mathbf{F}}({\mathbf{r}} - {\mathbf{R}})\cdot d{\mathbf{r}}.\end{array}$$

Here *ρ*^(*α*)^(**r**) is the free-molecule polarizability density as given in Eq. (1). The validity of this local approximation will be verified  by explicit comparison with the fully non-local response as shown below.

The Raman polarizability density, denoted as *δρ*^(*α*)^ = ∂*ρ*^(*α*)^/∂*Q*_*k*_, is the change of molecular polarizability density due to the vibrational mode *k*. It is calculated by the finite differentiation of polarizability densities with respect to small atomic displacements in a given vibrational mode. In a TERS junction, the effective Raman polarizability density is then represented as the free-molecule Raman polarizability density distributed over the near-field distribution (**F**(**r** − **R**)),

6 $$\delta \rho _{{\mathrm{loc}}}^{(\alpha )}({\mathbf{r}},{\mathbf{R}}) = {\mathbf{F}}({\mathbf{r}} - {\mathbf{R}})\cdot \delta \rho ^{(\alpha )}({\mathbf{r}})\cdot {\mathbf{F}}({\mathbf{r}} - {\mathbf{R}}).$$

The TERS intensity of a certain vibrational mode is proportional to the square of integrated effective Raman scattering polarizability density, formulated as

7 $$I(Q_k) \propto [{\int} \delta \rho _{{\mathrm{loc}}}^{(\alpha )}({\mathbf{r}})\cdot d{\mathbf{r}}]^2.$$

Due to the confinement of the near field, the integration over all space can be effectively approximated by local integration within a finite volume. This integration volume is determined by the full width at half maximum (FWHM) of the near-field distribution.

Here we have briefly summarized the method of LIRPD without explicitly writing down the element form of all matrices since only the *zz* component of the polarizability tensor is considered in calculating the Raman intensities (the long axis of the TERS junction aligns with the *z* direction). A detailed description of LIRPD in full tensor representation is provided in Supplementary Methods. The local approximation made in Eq. (3) can be improved by including the densities of higher-order polarizability tensors, for example, the quadrupole-dipole polarizability (${\cal{A}}$ tensor)^36^ density. It is equivalent of applying multipole expansion to the effective dipole operator^37,38^, which introduces a semi-local correction to the local approximation. Including ${\cal{A}}$-tensor densities slightly improves the accuracy when an atomically confined field is applied to a small molecule (benzene). But we find for larger systems, since the required near field is less confined, the contribution from ${\cal{A}}$-tensor densities becomes trivial. Therefore, all presented TERS images are calculated by considering only the dipole-dipole polarizability density. The TERS images with additional ${\cal{A}}$-tensor density contributions are provided for comparison in Supplementary Fig. 1.

**Fig. 1:**
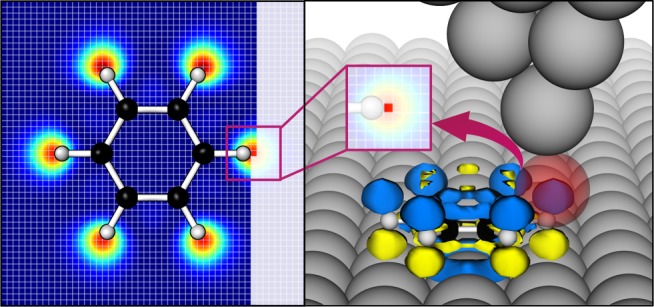
Schematic representation of locally integrated Raman polarizability density. The Raman polarizability density of the normal mode at 664 cm^−1^ of a benzene molecule is illustrated by isosurface (right panel). The density distributions in blue and in yellow hold the positive and negative signs, respectively. The red sphere denotes the confined near-field distribution at its FWHMs. The Raman polarizability densities in the red sphere are drastically enhanced and spatially integrated giving rise to a Raman intensity (symmetric bending) at 664 cm^−1^ (in insert). TERS image is generated from locally integrating Raman polarizability density by tip scanning over a benzene molecule (left panel)

Here we take a benzene molecule as an example to demonstrate how the LIRPD approach works for TERS imaging. The Raman polarizability density distribution is plotted on the right panel of Fig. 1. The positive value of density is colored blue and the negative value is in yellow. The near field is confined in a red sphere, which we call the effective integration volume. In this work the near-field distribution is expressed as a 3D Lorentzian function. Compared with the widely used Gaussian field model, Lorentzian distribution has slightly more pronounced tails, which better captures the background near field on the substrate away from the tip as is obtained from our atomistic electrodynamics calculations^15^. The diameter of the integration volume is the full width at half maximum (FWHM) of the field distribution. The Raman polarizability densities distributed within the red sphere are locally enhanced by the near field leading to the effective scattering polarizability densities, which are then integrated over all space to obtain Raman intensity that corresponds to the specific tip position recorded in the TERS image (Fig. 1 insert in the right panel). The imaging pattern is not sensitive to the field shape, which is shown in Supplementary Fig. 2. Without the confined near field, the integration of the Raman polarizability density over all space leads to the far-field Raman signals of the molecule. The mechanism of LIRPD explains the gradient-based selection rule in plasmon-enhanced Raman spectroscopy as well as the spatial localization of the TERS intensity.

### TERS imaging and selection rule

TERS images are obtained by scanning the tip over a sample molecule and simultaneously collecting the Raman signals. Atomically resolved TERS images were previously simulated, and the confinement of near field down to 5 Å in diameter was found necessary to achieve the ultrahigh resolution^26^. However, the local properties probed by the highly confined near field, which is key to establish the dependence of high-resolution TERS images on molecular normal modes, were  still not clear. For example, the simulated TERS images are drastically different between the symmetric and anti-symmetric bending modes of benzene, even though it is the same atoms that are vibrating in these two normal modes. Using the LIRPD method, we illustrate where such spatial variation originates and how it’s affected by the atomic vibrations. The consistency of this model is evidenced by reproducing the TERS images calculated by the hybrid atomistic electrodynamics/quantum mechanics method (DIM/QM) in ref. ^26^ which include the fully non-local response. Here we use the same symmetric and anti-symmetric bending modes of benzene as examples.

**Fig. 2:**
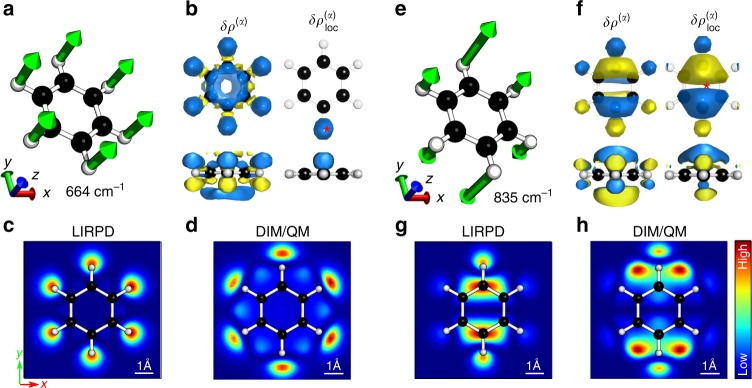
Normal modes, Raman polarizability densities, and TERS images of benzene. Normal modes **a**–**d** at 664 cm^−1^ and **e**–**h** at 835 cm^−1^. **a**, **e** Schematic representation of molecular vibrations. **b**, **f** Raman polarizability density (left column) and locally enhanced Raman polarizability density (right column) by a near field at the marked position (red asterisk) from the top view (top) and side view (bottom). The density is normalized and the absolute isovalue is set to 0.2 with the positive sign in blue and the negative sign in yellow. Simulated TERS images obtained by LIRPD in **c**, **g**, and the hybrid atomistic DIM/QM in **d**, **h**, respectively

The normal modes of the symmetric (Fig. 2a) and anti-symmetric bending vibrations (Fig. 2e) and the related Raman polarizability density distributions (Fig. 2b, f) were calculated with the molecule-substrate mutual polarization taken into account. The spatial distributions of the Raman polarizability densities and the molecular vibrations are highly correlated. In the 664 cm^−1^ mode, all the hydrogen atoms symmetrically bend out of the molecular plane. The corresponding density distribution preserves the symmetry. The densities are largely localized on the hydrogen atoms and benzene ring (top of Fig. 2b). The distribution is symmetric, but the signs are opposite with respect to the molecular plane (bottom of Fig. 2b). The large atomic displacement leads to the prominent density distributions on the hydrogen atoms. The densities distributed over the benzene ring come from the coupled motions of the carbon atoms. The 835 cm^−1^ mode is featured by anti-symmetric out-of-plane vibrations (Fig. 2e). The corresponding density distribution inherits the same anti-symmetry by having opposite signs in *xy* plane either above or below the molecular plane. The in-plane opposite signs stem from the para-hydrogen atoms coupled with the attached carbon atoms vibrating in opposite directions. Across the molecular plane, the densities also have opposite signs around the same atoms.

The near field is here represented by a 3D Lorentzian distribution with FWHMs of 1.3 Å centered at 1.0 Å above the benzene plane in our simulations. By using Eq. (6), the Raman polarizability densities are enhanced within the Lorentzian peak, while the densities outside the peak are smeared out. In such way the Raman density distribution is extracted from a small volume defined by the confined near field. In other words, the Raman densities are locally selected by the confined near field. The selected densities are then integrated over space to obtain a Raman intensity. For the mode at 664 cm^−1^, the densities at the near-field position rather than elsewhere are greatly boosted. The integrated density (local polarizability density) is largely due to the densities with the same sign being accumulated, resulting in a strong TERS signal. In contrast, the integrated density is close to zero in the integration volume above the center of benzene, because the local densities are distributed with opposite signs and thus are integrated to zero. We accordingly see a quite low intensity in the center of the TERS image at 835 cm^−1^.

Using the LIRPD method to calculate Raman intensities while scanning the tip over a molecule, we are able to reproduce the high-resolution TERS images predicted by the DIM/QM method (Fig. 2c, d, g, h). In general, the TERS intensity is predominantly determined by two factors: the Raman polarizability density distribution and the local integration volume (near-field distribution) in terms of size and position. The Raman polarizability density distribution is dominated by the large atomic displacements in a normal mode, and governs the pattern of its TERS image. A narrow near-field distribution leads to the atomic resolution in TERS images. For instance, the image resolution is sensitive to field FWHMs of the *x* and *y* components rather than the *z* component. Moreover, the height from tip to molecular plane, which is similarly defined as the near-field focal plane in ref. ^26^, plays a vital role in TERS imaging. A small change of tip height leads to a significantly different TERS image. These findings suggest that distributing the near fields within atomic dimensions over an appropriate imaging plane is the key to the atomic resolution in TERS images. (see Supplementary Fig. 3).

It is noted that the integration of the Raman polarizability density without the confined near field leads to the typical far-field property of the molecule. For the selected two modes of benzene, the Raman polarizability density distributions are symmetric with opposite signs so that integration over all space is zero. This means the Raman signals are silent for the specific modes, which is consistent with the traditional selection rules. However, the confined near field breaks the symmetry, and thus leads to non-zero values after the integration. It provides the explanation for the inactive Raman modes being evoked in plasmon-enhanced Raman spectra. This symmetry breaking of Raman polarizability density distribution aligns with the field-gradient effects typically invoked to explain the high spacial resolution^15,26,39^. In TERS images, the hotspots indicate the tip positions locally break the symmetry.

### Complex Raman polarizability density in resonant TERS

**Fig. 3:**
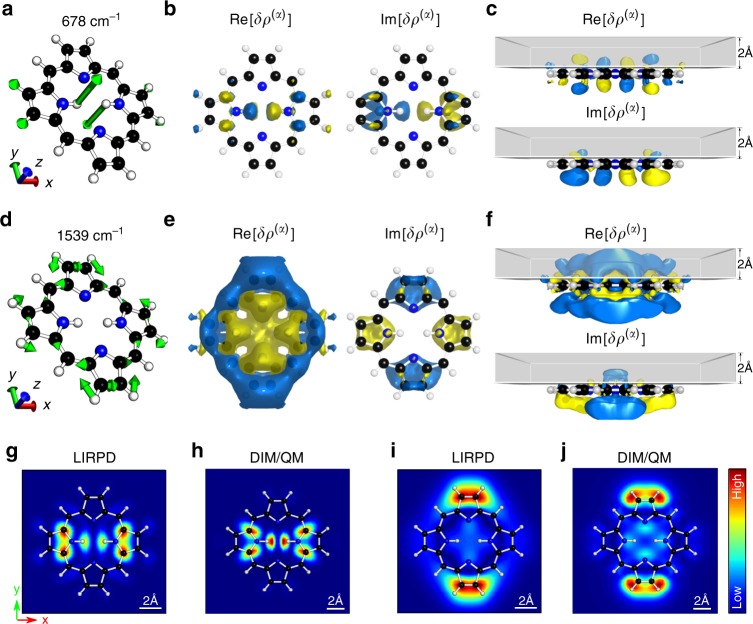
Normal modes, Raman polarizability densities, and TERS images of porphyrin. **a**–**c** Normal modes at 678 cm^−1^ and **d**–**f** at 1539 cm^−1^. **a**, **d** Schematic representation of molecular vibrations. **b**, **e** The Raman polarizability densities in real (left) and imaginary (right) parts of porphyrin on a Au(111) surface from the top view. **c**, **f** The Raman polarizability densities are distributed throughout the scanning volume with the thickness of 2 Å above porphyrin plane from the side view. The densities are normalized and the absolute isovalue is set to 0.2 with the positive sign in blue and the negative sign in yellow. Simulated TERS images of modes at 678 cm^−1^ (**g**, **h**) and 1539 cm^−1^ (**i**, **j**) obtained by LIRPD (**g**, **i**) and DIM/QM approaches (**h**, **j**), respectively

The LIRPD model is naturally transferable to resonant TERS spectra. Contributions from both the electronic and the vibrational transitions are coherently included in the Raman polarizability, which now has a non-trivial imaginary part. We take free-base porphyrin as an example to explore the correlation between Raman polarizability densities and resonant TERS images. Two representative modes of porphyrin are selected: one out-of-plane vibrational mode at 678 cm^−1^ and one in-plane mode at 1539 cm^−1^. The 678 cm^−1^ mode is characterized by the opposite out-of-plane bending of two hydrogen atoms attached to the para-nitrogens (Fig. 3a). The applied excitation energy is at 2.29 eV corresponding to the Q_*y*_(0, 0) transition of porphyrin.

As shown in Fig. 3b, c, the real part of the density distribution reflects the dominant atomic displacement, and is symmetrically distributed with respect to the molecular plane with opposite signs. In the 678 cm^−1^ mode, the atoms vibrate perpendicularly to the molecular plane. Similar to the benzene out-of-plane modes, the real Raman polarizability density here is distributed closely around the vibrating atoms, and has opposite signs above and below the molecular plane. In contrast, the imaginary density distribution is asymmetric with respect to the molecular plane, where most of the densities are distributed underneath the porphyrin molecule. However, the imaginary Raman density distribution preserves the same symmetry as in the vibrational mode underneath the molecule. Similar trend is also observed in the 1539 cm^−1^ mode (Fig. 3e, f). As 1539 cm^−1^ mode is an in-plane mode, the real Raman polarizability densities are more broadly distributed in-plane. The direction from positive to negative values follows the overall trend of the atomic displacement.

By locally integrating the complex Raman polarizability densities enhanced by the near field, mode-specific resonant TERS images with atomic resolution are obtained (Fig. 3g–j). The near fields with FWHMs of 2 Å are placed 1.5 Å above the molecular plane. The effective densities distributed in the scanning volume are illustrated in Fig. 3c, f and the locally enhanced Raman polarizability densities by the given tips are given in Supplementary Fig. 4. We again see the strong resonant Raman intensities at the dominant atomic displacements, which is consistent with the atomistic simulation results (Fig. 3g–j). The patterns in TERS images are mostly similar to the real Raman polarizability density distributions, which is attributed to the facts that the imaginary part of the density is overall much weaker than the real part and that the imaginary Raman densities are largely distributed underneath the molecule. The weak imaginary *zz* polarizability is expected, because the Q_*y*_(0, 0) band has a very weak oscillator strength due to the transition dipole moment on the *xy* plane. The mutual polarization dominates the interaction between the molecule and the substrate, which explains the imaginary Raman polarizability densities underneath the molecule. The overall patterns in these Raman polarizability density distributions and the corresponding TERS images align with the electronic transition dipole moment, which is along *y* axis in this specific example. We note that the TERS image of the 1539 cm^−1^ mode is not intuitively correlated to the real density distribution shown in Fig. 3e. Actually, the maximal density corresponding to the brighter hotspots in the TERS image is four-fold larger than the densities around the nitrogen atoms. This density value difference explains the contrast in the TERS image.

Self-consistent solutions^26,27,30^ of molecular property perturbed by a confined near field are considered the most accurate at the TDDFT level of theory, as they calculate the fully non-local response of the molecule to the near field (Eq. (4)). DIM/QM is regarded as benchmark in this work, because it provides a consistent treatment of both the near-field distribution and the molecular properties. The local approximation made in Eq. (5) qualitatively reproduces the results from DIM/QM, which is an evidence of the validity of LIRPD approach. The agreement between the LIRPD and DIM/QM results is qualitatively good. Because while the overall symmetry patterns of the benzene TERS images are reserved in LIRPD, the two key features for benzene are also captured: hotspots are slightly off the atoms, and Raman inactive modes are activated by strong field gradient (Supplementary Fig. 5). Because of this local treatment of the electronic density, the FWHM of the Lorentzian field has to be smaller than DIM/QM. The agreement between the LIRPD and DIM/QM results can be improved by considering the multipole expansion at the density level. In Supplementary Fig. 1, we have shown that for small molecules like benzene and porphyrin, the ${\cal{A}}$-tensor densities drives the hotspots slightly further away from the vibrating atoms, and the effective integration volume becomes closer to that in the DIM/QM simulations. However, the contribution from ${\cal{A}}$-tensor decreases when the near field confinement is beyond the atomic scale, which is the case for the following analysis regarding interpreting experimental results. Moreover, because the Raman polarizability density in LIRPD is independent on the tip position, the LIRPD calculation is order of magnitude (the total number of grids) faster than DIM/QM, which is advantageous for analyzing large molecules seen in experiments.

### Interpreting experimental TERS images

In the pioneering work of TERS imaging^24^, a single molecule of *meso*-tetrakis(3,5-di-*tert*-butylphenyl)porphyrin (H_2_TBPP) was visualized with sub-nanometer resolution via precise tuning of the plasmon resonance coupled with molecular vibrations. The four-fold symmetry in both experimental and simulated TERS images are invariant across different normal modes, which was attributed to electronic resonance and tautomerization^24,27^. Using the energetically favored concave configuration of H_2_TBPP^23,27^, we find that the strong interaction between molecule and the silver substrate leads to more than 100 nm red shift of the Q and B bands in the absorption spectra in Supplementary Fig. 6. Hence it is questionable to assume the free H_2_TBPP excitation in resonant TERS simulations. In our simulations we took the polarization interactions between the molecule and the metal substrate into account, and found that the B_*x*_(0, 0) and B_*y*_(0, 0) transitions of H_2_TBPP is excited at around 560 nm, while Q_*y*_(0, 0) band is excited at 760 nm (Supplementary Table 1). So the 532 nm laser used in the experiment is more likely to excite the B-band transition of H_2_TBPP. Therefore, we revisit the resonant TERS imaging of H_2_TBPP, and interpret the invariant patterns across different normal modes based on the LIRPD mechanism.

**Fig. 4:**
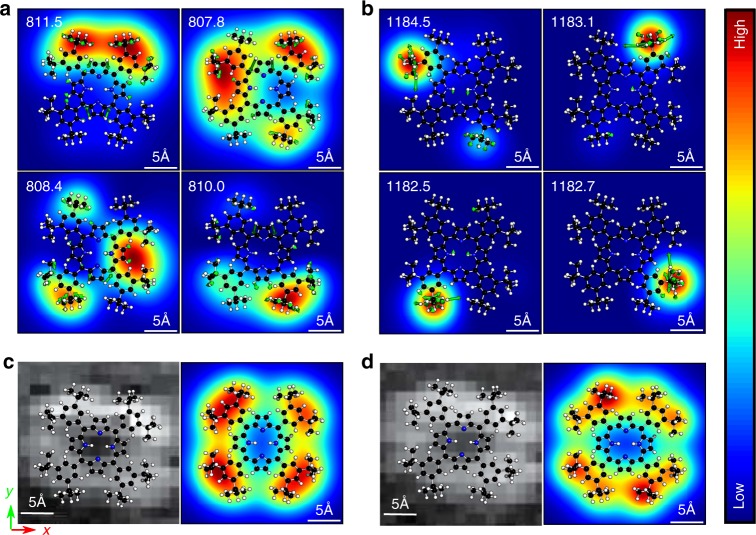
The resonant TERS images of H_2_TBPP molecule absorbed on a Ag(111) surface. The excitation energy corresponds to the B_*y*_ transition. **a**, **b** TERS images of individual degenerate modes of the bands centered around 810 and 1185 cm^−1^. The schematic representations of molecular vibrations lay on the individual TERS images and the corresponding frequencies (in cm^−1^) are given in the top left corners. **c**, **d** The experimental TERS images (left panel) and simulated TERS images by integrating band width of 20 cm^−1^ (right panel)

In order to well describe the near-field distributions in plasmonic junction, the correlation between gap size and near-field confinement was investigated. The details are provided in Supplementary Table 2. A reasonable approximation to the plasmonic near field is that FWHMs are 12 Å for the *x* and *y* components and 6.0 Å for the *z* component. The narrower distribution along *z*-axis is due to the fact that the near field is squeezed by the short-range dipole-dipole interaction in the nanocavity^15,40^. The center of this field distribution is placed 2.7 Å above the molecule. The TERS spectrum obtained by LIRPD with the field center on top of a lobe agrees well with experimental spectrum (Supplementary Fig. 7). Based on the simulated TERS spectrum, the TERS mapping at the critical spectral peaks were elaborately explored. We find that there exists multiple degenerate modes within the integration window in ref. ^24^, and each of these modes has a distinct TERS image. The modes with the largest TERS intensities are featured by prominent butyl vibrations. For example, the region around 810 cm^−1^ is associated with the modes at 807.8, 808.4, 810.0, and 811.5 cm^−1^, which are characterized by the vibrations of different butyl groups (Fig. 4a). The simulated TERS images with B_*y*_(0, 0) excitation are shown in Fig. 4. Combining the TERS images of the dominant modes around 810 cm^−1^ within a 20 cm^−1^ band width, we find the total TERS image matches the experimental mapping (Fig. 4c), also exhibiting the four-lobe symmetric pattern covering the butyl groups. Since the integration volume is above the entire molecule, the pyrrole vibrations are not captured in the TERS image (see Supplementary Fig. 8). The combined TERS image at around 1185 cm^−1^ is similar to 810 cm^−1^. The simulated four-lobe pattern matches the experimental mapping (Fig. 4d), and the multiple modes featured by butyl vibrations dominantly contribute to the TERS image (Fig. 4b). The TERS images at frequencies at 900, 990, and 1520 cm^−1^ are simulated as well (Supplementary Information Fig. 9), and all are consistent with the experimental results. Particularly, the contrast and central dark area becomes smaller toward the high wavenumbers in our simulations.

The four-fold symmetry in H_2_TBPP TERS images was previously attributed to hydrogen tautomerization^26,27^. However, in this work we clearly see that the four-fold symmetry is obtained by combining the TERS images of degenerate modes, without tautomer contributions. The degenerate vibrations comes from the symmetry of the molecular structure. In the experiment reported in ref. ^24^, it is very likely that all the four degenerate modes are included in the integration window, which leads to the same four-fold symmetry across different frequency regions. By enforcing tautomerization, the TERS images remain the same except being slightly more smooth and symmetric (Supplementary Fig. 10). Thus, we believe that TERS images of H_2_TBPP are not sensitive to hydrogen tautomerization. We will further discuss the tautomerization effect on TERS images using a porphycene molecule whose tautomers have been clearly identified in STM experiments.

Moreover, we find the TERS images calculated at Q_*y*_(0, 0) and B_*y*_(0, 0) transitions are almost identical, as shown in Supplementary Fig. 11. This suggests that the Raman scattering properties of the side groups, which dominates the TERS images, are insensitive to these excited states. This is expected because both of these electronic excitations are localized in the base porphyrin ring. The TERS tip won’t be able to probe the base ring unless is forced down to the bottom of the molecule.

It is generally difficult to differentiate H_2_TBPP normal modes based on TERS images, as was seen in experiment. Our simulation results suggest that the prevailing four-fold symmetry in H_2_TBPP TERS images is largely due to the combination of multiple degenerate modes with butyl vibrations, rather than tautomerization or electronic resonance effects. One would expect the TERS images of H_2_TBPP to be more differentiable if higher spatial resolution is achieved in experiments, and if more precise Raman measurements are performed so that the integration window becomes narrower to eliminate multiple mode contributions. Nevertheless, the LIRPD method offers a consistent and flexible approach to the interpretation of experimental measurements on large molecules.

### TERS imaging for microscopic structure characterization

We further explore the effect of hydrogen tautomerization on TERS images, and at the same time demonstrate how TERS imaging can be applied as a structural characterization tool. We take porphycene as an example, of which the tautomers have been identified in experiment with the help of low-temperature STM^41^. The optimized geometries of three porphycene tautomers, one *trans* and two *cis* configurations (denoted as *cis* and *cis*′), are shown in Supplementary Fig. 12. The *trans* and *cis* porphycene are planar, while the hydrogen atoms in the cavity of *cis*′ porphycene are out of the macrocycle plane due to a strong steric repulsion. In the TERS simulation using the LIRPD methods, we examine the normal mode around 1250 cm^−1^, as it was previously reported to be a prominent peak in resonant SERS^42^. The near field is represented in 3D Lorentzian distribution with the FWHM of 5 Å for all three Cartesian components and is centered at 2 Å above the molecule.

**Fig. 5:**
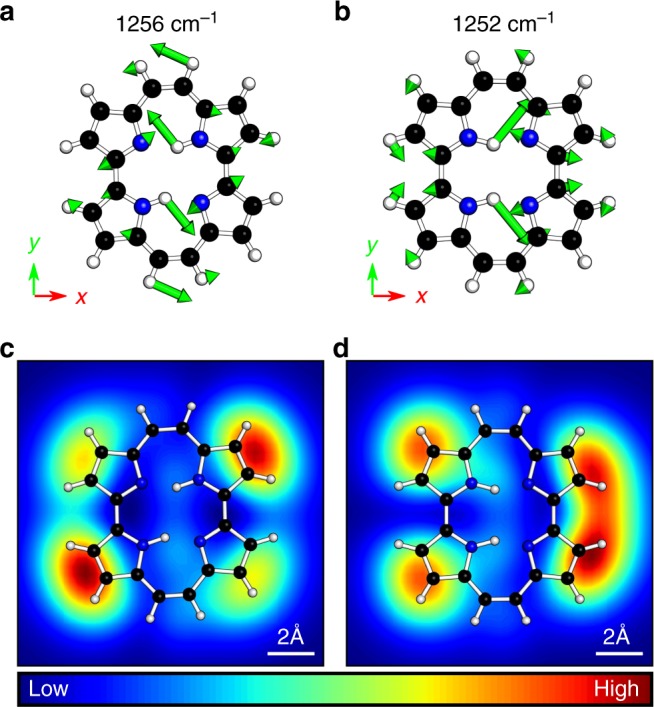
Normal modes and TERS images of porphycene tautomers. Normal modes **a** at 1256 cm^−1^ and **b** at 1252 cm^−1^ make the dominant contributions to the TERS images at ~1250 cm^−1^ of **c** *trans* and **d** *cis* tautomers adsorbed on a Cu(110) surface, respectively

The resonant TERS images generated by the LIRPD method at the excitation energy of 2.21 eV are shown in Fig. 5. The simulations suggest that two modes contribute to the total TERS image at 1250 cm^−1^ with the band width of 20 cm^−1^. The dominant mode for each tautomer is characterized by central hydrogen atoms vibrations coupled with pyrrole moieties (Fig. 5a, b). The Raman polarizability density distributions of the individual modes within the scanning volumes are illustrated in Supplementary Fig. 13. We again see the resonant TERS image is largely determined by the real densities. The *para*-hydrogen atoms vibrating oppositely in the cavity lead to the large density distributions on the *para*-pyrrole moieties in the *trans* configuration. In the *cis* configuration the prominent density distributions are related to the *ortho*-hydrogen vibrations. The modes with the large displacement of the central hydrogen atoms provide the major contributions to the total TERS images. Generally, the overall hotspot symmetry follows the configuration of the two central hydrogens. There are four hotspots with one brighter pair on *para*-pyrrole moieties for the *trans* configuration. For the *cis* configuration, there are two connected hotspots on the adjacent pyrrole and separate lobes on the other two pyrrole moieties. The TERS image of the *cis*′ configuration was simulated as well (Supplementary Fig. 14).

The simulations indicate that different tautomers can be identified and differentiated through distinct TERS images, and the patterns are either *trans* or *cis* following the configuration of the central hydrogens. TERS imaging carries both structural and chemical information of mode vibrations, and TERS images can be even more distinguishable among tautomers. Thus, by combining the LIRPD interpretation with high-resolution measurements, we envision TERS to be complementary to STM for microscopic characterization.

## Discussion

In this work we illustrated that high-resolution TERS probes the molecule’s local polarizability density changes. The Raman polarizability densities are locally enhanced by the confined near field and then integrated over space giving rise to the molecule’s near-field response. The density distribution is unique for specific normal mode, and leads to the also unique TERS image. The local symmetry breaking in the integrated density distribution is the root of the spatial variation of TERS intensities, and explains the gradient-based selection rules in TERS. The locally integrated Raman polarizability density provides theoretical insights into experimental TERS images and origin of the hotspots from a point of view of the molecule’s locally probed property. The LIRPD mechanism is a simple and intuitive approach to the interpretation of high-resolution TERS images. With the help of LIRPD interpretation, we demonstrated that TERS imaging can be applied to resolve subtle changes in molecular structure with atomic precision.

The key to achieve the atomistic resolution is to confine the near field down to a few Ångstroms in experiment. Previous simulations indicate that such confinement requires the tip to be atomically sharp^28,40,43–45^. To maintain a stable sharp tip during the scanning, cryogenic and high-vacuum environment is preferred in experiments^15,46^. Moreover, this work and previous simulations^26^ suggest that the TERS images are also very sensitive to the height of the focal plane relative to the molecule and the field confinement in the vertical axis, and thus flat molecules are generally favored. All these conditions to obtain high-resolution TERS images are difficult to fulfill. However, we still expect TERS imaging has the potential to rival with state-of-the-art scanning tunneling microscopy for microscopic characterization, and thus holds great promise for monitoring chemical structure and transformation with sub-molecular resolution.

## Methods

### DIM/QM calculations

A locally modified version of the Amsterdam Density Functional (ADF) program package^47–49^ was employed to perform all the simulations. The geometry optimizations, frequency, and linear-response calculations were carried out using the Becke–Perdew (BP86) exchange-correlation functional with the triple-*ζ* polarized (TZP) Slater-type basis, except for the H_2_TBPP molecule, which was calculated at the BP86/DZP theoretical level in order to reduce the computational cost. The geometries of benzene and porphyrin molecules were optimized with small frozen core in the absence of metal substrate to be consistent with conditions of the previous work^26^. The adsorbed structures of H_2_TBPP and the porphycene molecules are strongly influenced by the molecule-substrate interactions. The metal substrate was included in the geometry optimizations accordingly.

### Polarizability density calculations

The excited state lifetime is set to 0.1 eV^50^ and the metal substrates which are large enough to support a sample molecule were treated with the discrete interaction model (DIM)^51^. The frequency-dependent complex dielectric functions of metal surface were obtained from Johnson and Christy^52^. The cubic grids used for representing the density are determined by the sample molecule structure and orientation on the surface. The boundary of the box is 4 Å away from a H_2_TBPP molecule and 3 Å for the other molecules. The step size is 0.4 Å for generating grids parallel to the metal surface and 0.2 Å for H_2_TBPP and 0.1 Å for others in the vertical direction. The Raman polarizability densities are obtained by the three-point numerical differentiation method. The Raman polarizability densities are locally enhanced by near fields and are locally integrated. From the locally integrated Raman polarizability density, differential cross section (d*σ*/dΩ) of Raman scattering is written as

8 $$\frac{{{\mathrm{d}}\sigma }}{{{\mathrm{d}}\Omega }} = \frac{{\pi ^2}}{{\epsilon _0^2}}(\tilde \nu _{{\mathrm{in}}} - \tilde \nu _k)^4\frac{h}{{8\pi ^2c\tilde \nu _k}}\frac{{|\alpha_k^\prime |^2}}{{1 - {\mathrm{exp}}( - hc\tilde \nu _k/k_{\mathrm{B}}T)}},$$

where $\tilde \nu _{{\mathrm{in}}}$ is the incident frequency and $\tilde \nu _k$ is the frequency of *k*^*th*^ normal mode. $\alpha _k^\prime$ is the locally integrated Raman polarizability density related to polarizability density of *k*^*th*^ normal mode. Here we considered only *zz* component contributes to TERS cross sections, and the temperature was set to 298 K.

## Supplementary information

Supplementary Information

## Data Availability

The Raman polarizability densities used to generate TERS images are available upon request. Exemplar data are provided along with the source code repository. The LIRPD code is available at https://github.com/jensengrouppsu/LIRPD.
