# Supplementary material for: High-resolution tip-enhanced Raman scattering probes sub-molecular density changes
Source: Nat Commun. 2019 Jun 12;10:2567. doi: 10.1038/s41467-019-10618-x (PMC6561954; doi:10.1038/s41467-019-10618-x)
Supplement: Supplementary file 1 — Supplementary Information [file 41467_2019_10618_MOESM1_ESM.pdf]

Supplementary Information
High-resolution tip-enhanced Raman scattering probes sub-molecular density changes

Chen et al.

Supplementary Methods

Local approximation and electric multipole expansion. Within the framework of linear-response time-dependent density functional theory, we are interested in calculating the molecule's polarizability due to an electric field perturbation. The molecule's first-order induced charge density ($\delta\rho$) due to the perturbation and its polarizability (α) are obtained using linear-response time-dependent density functional theory (LR-TDDFT), which is given by

$$\delta\rho_\alpha(\mathbf{r},\omega) = \int \chi(\mathbf{r},\mathbf{r}',\omega)\hat{v}^{\text{pert}}_\alpha(\mathbf{r}',\omega)d\mathbf{r}' \ , \tag{1}$$

$$\alpha_{\alpha\beta}(\omega) = \int \hat{\mu}^{\text{eff}}_\alpha(\mathbf{r},\omega)\delta\rho_\beta(\mathbf{r})d\mathbf{r} \ = \iint \hat{\mu}^{\text{eff}}_\alpha(\mathbf{r},\omega)\chi(\mathbf{r},\mathbf{r}',\omega)\hat{v}^{\text{pert}}_\beta(\mathbf{r}',\omega)d\mathbf{r}'d\mathbf{r} \ . \tag{2}$$

In these two equations, the subscripts (α, β, and other Greek indices hereafter) indicate the Cartesian direction, $\mathbf{r}$ is an arbitrary vector denoting the position in space, and the equation is Fourier transformed into the frequency (ω) domain. The density-density response function, $\chi(\mathbf{r},\mathbf{r}',\omega)$, is given by[1]

$$\chi(\mathbf{r},\mathbf{r}',\omega) = \lim_{\eta\to 0}\sum_m \frac{\langle 0|\hat{n}(\mathbf{r})|m\rangle\,\langle m|\hat{n}(\mathbf{r}')|0\rangle}{\omega-\omega_{m0}+\mathrm{i}\eta} - \frac{\langle 0|\hat{n}(\mathbf{r}')|m\rangle\,\langle m|\hat{n}(\mathbf{r})|0\rangle}{\omega+\omega_{m0}+\mathrm{i}\eta} \ , \tag{3}$$

where m is the sum over all excited states of the system, ω_{m0} is the excitation energy, $\hat{n}(\mathbf{r})$ is the density operator, and η is the damping factor. The perturbation operator, $\hat{v}^{\text{pert}}_\beta(\mathbf{r}',\omega)$ accounting for the electric field perturbation polarized in β direction is given by

$$\hat{v}^{\text{pert}}_\beta(\mathbf{r}'-\mathbf{R}) = -(\delta_{\beta\delta} + F^\beta_\delta(\mathbf{r}'-\mathbf{R}))\cdot\hat{\mu}_\delta(\mathbf{r}') \ , \tag{4}$$

where $\hat{\mu}_\beta(\mathbf{r}')$ is the dipole moment operator. Here $\delta_{\beta\delta}$ is the Kronecker delta function, and F^β_δ represents the plasmonic local field in δ direction due to the external field polarized in β direction. For molecules in a TERS junction, $\hat{v}^{\text{pert}}$ is dominated by the plasmonic near field that is localized around the tip position $\mathbf{R}$. Thus this operator becomes also a function of

$\mathbf{R}$. The effective dipole operator, $\hat{\mu}_{\alpha}^{\text{eff}}(\mathbf{r},\omega)$, describes the radiation of oscillating dipoles,[2,3] and can be expanded in the electric multipole format.

$$\hat{\mu}_{\alpha}^{\text{eff}}(\mathbf{r}) \approx -\big[\delta_{\alpha\gamma} + F_{\gamma}^{\alpha}(\mathbf{r}-\mathbf{R})\big]\hat{\mu}_{\gamma} - \frac{1}{3}F_{\gamma\epsilon}^{\alpha}(\mathbf{r}-\mathbf{R})\hat{\theta}_{\gamma\epsilon}\ . \tag{5}$$

$F_{\gamma(\epsilon)}^{\alpha}(\mathbf{r}-\mathbf{R})$ is the local field (gradient) distribution in the $\gamma(\epsilon)$ direction that radiates a field polarized in α direction.[3] $\hat{\mu}_{\gamma}$ is the dipole operator and $\hat{\theta}_{\gamma\epsilon}$ is the traceless quadrupole operator. All the molecular properties and near fields are dependent on the frequency (ω) of the external field and generally complex. In the following we drop the ω notation for simplicity. The polarizability in the presence of the tip is then given by

$$\alpha_{\alpha\beta}(\mathbf{R}) = \int \left\{ \big[\delta_{\alpha\gamma} + F_{\gamma}^{\alpha}(\mathbf{r}-\mathbf{R})\big]\hat{\mu}_{\gamma} - \frac{1}{3}F_{\gamma\epsilon}^{\alpha}(\mathbf{r}-\mathbf{R})\hat{\theta}_{\gamma\epsilon} \right\} \int \chi(\mathbf{r},\mathbf{r}')\hat{\mu}_{\delta}\big[\delta_{\beta\delta} + F_{\delta}^{\beta}(\mathbf{r}'-\mathbf{R})\big]d\mathbf{r}'d\mathbf{r}\ . \tag{6}$$

Ideally, $\alpha_{\alpha\beta}$ would be obtained self-consistently in the presence of the confined near field.[4–6] This is what is done in the DIM/QM method. But in doing so, the computational cost scales up with the number of grid points necessary for the tip scanning to cover the whole molecule, which becomes nearly infeasible for large molecules like porphyrin complexes. More importantly, it doesn't allow for decomposing the molecular property into atomic contributions. To simultaneously address these two problems, we make a local approximation to polarizability in Supplementary Equation 6. Since both $F_{\gamma}^{\alpha}(\mathbf{r}-\mathbf{R})$ and $F_{\gamma}^{\alpha}(\mathbf{r}'-\mathbf{R})$ are localized around the tip-position $\mathbf{R}$ we expect that the largest contributions to the response comes from $F_{\gamma}^{\alpha}(\mathbf{r}-\mathbf{R}) \sim F_{\gamma}^{\alpha}(\mathbf{r}'-\mathbf{R})$. Making this local approximation for the polarizability we

obtain

$$\begin{aligned}
\alpha_{\alpha\beta}(\mathbf{R}) &\overset{\text{local}}{\approx} \int \left\{ \left[\delta_{\alpha\gamma} + F^{\alpha}_{\gamma}(\mathbf{r}-\mathbf{R})\right]\hat{\mu}_{\gamma} + \frac{1}{3}F^{\alpha}_{\gamma\epsilon}(\mathbf{r}-\mathbf{R})\hat{\theta}_{\gamma\epsilon} \right\} \cdot \delta\rho^{\text{free}}_{\delta}(\mathbf{r}) \cdot \left[\delta_{\beta\delta} + F^{\beta}_{\delta}(\mathbf{r}-\mathbf{R})\right] d\mathbf{r} \\
&= \int \left[\delta_{\alpha\gamma} + F^{\alpha}_{\gamma}(\mathbf{r}-\mathbf{R})\right] \cdot \hat{\mu}_{\gamma}\delta\rho^{\text{free}}_{\delta}(\mathbf{r}) \cdot \left[\delta_{\beta\delta} + F^{\beta}_{\delta}(\mathbf{r}-\mathbf{R})\right] d\mathbf{r} \\
&\quad + \int \frac{1}{3}F^{\alpha}_{\gamma\epsilon}(\mathbf{r}-\mathbf{R}) \cdot \hat{\theta}_{\gamma\epsilon}\delta\rho^{\text{free}}_{\delta}(\mathbf{r}) \cdot \left[\delta_{\beta\delta} + F^{\beta}_{\delta}(\mathbf{r}-\mathbf{R})\right] d\mathbf{r} \\
&= \int \left[\delta_{\alpha\gamma} + F^{\alpha}_{\gamma}(\mathbf{r}-\mathbf{R})\right] \cdot \rho^{(\alpha)}_{\gamma\delta}(\mathbf{r}) \cdot \left[\delta_{\beta\delta} + F^{\beta}_{\delta}(\mathbf{r}-\mathbf{R})\right] d\mathbf{r} \\
&\quad + \int \frac{1}{3}F^{\alpha}_{\gamma\epsilon}(\mathbf{r}-\mathbf{R}) \cdot \rho^{(\mathcal{A})}_{\gamma\epsilon,\delta}(\mathbf{r}) \cdot \left[\delta_{\beta\delta} + F^{\beta}_{\delta}(\mathbf{r}-\mathbf{R})\right] d\mathbf{r}\ .
\end{aligned} \tag{7}$$

The Einstein summation convention is employed for repeated Greek indices. In Supplementary Equation 7 two polarizability densities are defined. $\rho^{(\alpha)}_{\gamma\delta}$ is the density of the free-molecule dipole-dipole polarizability, or alpha density. $\rho^{(\mathcal{A})}_{\alpha\gamma\beta}$ is the density of the free-molecule quadrupole-dipole polarizability, or $\mathcal{A}$-tensor density. These two terms account for the radiating dipoles and quadrupoles of the molecule. Definitions of the molecular multipole polarizabilies can be found in ref. 7. As discussed above these molecular densities should have been solved in the presence of the localized near-field around the tip, however, in the local approximation we assume that these are obtained as the response to a uniform electric field. This neglects the nonlocal coupling in the linear response that is present in the full DIM/QM simulations and thus cannot be expected to describe the full response. In this work the usefulness of this local approximation is carefully benchmarked against the full DIM/QM response as shown in the main text. In this work, we consider only the zz component of the polarizability, which is written as

$$\begin{aligned}
\alpha_{zz}(\mathbf{R}) = &\int \left[1 + F^{z}_{z}(\mathbf{r}-\mathbf{R})\right] \cdot \rho^{(\alpha)}_{zz}(\mathbf{r}) \cdot \left[1 + F^{z}_{z}(\mathbf{r}-\mathbf{R})\right] d\mathbf{r} \\
&+ \int \frac{1}{3}F^{z}_{z\epsilon}(\mathbf{r}-\mathbf{R}) \cdot \rho^{(\mathcal{A})}_{z\epsilon,z}(\mathbf{r}) \cdot \left[1 + F^{z}_{z}(\mathbf{r}-\mathbf{R})\right] d\mathbf{r}\ .
\end{aligned} \tag{8}$$

It is noted that integrating these densities over all space reproduces the corresponding

molecular α tensor and $\mathcal{A}$ tensor in the dressed-tensors formalism.[8,9] The difference between LIRPD and the dressed-tensors formalism lies in the multipole expansion of the perturbation operator. For the full dressed-tensors formalism, the dipole-quadrupole (A tensor) and quadrupole-quadrupole(C tensor) are typically used in addition to the α and $\mathcal{A}$ tensors. The A and C tensors describe the molecular dipole and quadrupole induced by the gradient of the near field. This is equivalent to a multipole expansion of the perturbation operator $\hat{v}^{\text{pert}}$ in the calculation of induced charge density. The full dressed-tensors method can reproduce the benchmark spectra,[3,4,10] under two assumptions: 1) the field gradient is small; 2) the field distribution has a fixed center. These two assumptions ensure that the leading term in the multipole expansion is the dipole-dipole term, and that the expansion point for A and C tensors is the same as the field gradient. However, these two assumptions break down for the highly confined near field in TERS. Therefore, the use of A and C tensors as was in the dressed-tensors formalism does not correctly describe TERS imaging.

The Raman polarizability densities ($\delta\rho^{(\alpha)}$) are the derivatives of polarizability densities with respect to the mass-weighted normal modes, which were calculated using the three-point numerical differentiation approach.

$$\delta\rho^{(\alpha)}(\mathbf{r}) = \frac{\partial\rho^{(\alpha)}(\mathbf{r})}{\partial Q_k} = \frac{\rho^{(\alpha)}(Q_k + \Delta Q_k) - \rho^{(\alpha)}(Q_k - \Delta Q_k)}{2 s_Q \Delta Q_k} \ , \tag{9}$$

where s_Q is the mass-weighted step size. The polarizability densities were calculated at the equilibrium geometry distorted along the normal mode coordinates in positive and negative directions. The zz components of the locally enhanced Raman polarizability densities ($\delta\rho^{(\alpha)}_{\text{loc}}(\mathbf{r})$), which are plotted for benzene and porphyrin in main text Figure 3 and 4, are obtained by replacing $\rho^{(\alpha)}$ in Supplementary Equation 7 with $\delta\rho^{(\alpha)}$. It is written as,

$$\delta\rho^{(\alpha)}_{\text{loc}}(\mathbf{r}) = \left[1 + F^z_z(\mathbf{r} - \mathbf{R})\right] \cdot \delta\rho^{(\alpha)}_{zz}(\mathbf{r}) \cdot \left[1 + F^z_z(\mathbf{r} - \mathbf{R})\right] \ . \tag{10}$$

When the near-field confinement is beyond atomic scale, the alpha density is the domi-

nant term. The use of $\mathcal{A}$-tensor density improves the imaging for small flat molecules, but its effect becomes insignificant for non-planar large molecules or unconfined field. Higher order terms, *e.g.*, octupole density, should lead to increasingly accurate reproduction of the self-consistent polarizability. We will show in Supplementary Figure 1 that good accuracy is achieved by considering only the first term in Supplementary Equation 7.

Lorentzian model of the near field distribution. In our simulations, the plasmonic local field is represented by a 3D Lorentzian function, which is given as

$$\begin{aligned} F^{\text{loc}} &= M\Big[\big(\frac{x-a}{\frac{1}{2}\tau_x}\big)^2 + \big(\frac{y-b}{\frac{1}{2}\tau_y}\big)^2 + \big(\frac{z-c}{\frac{1}{2}\tau_z}\big)^2 + 1\Big]^{-1} \\ &\quad + N\Big[\big(\frac{x-a}{\frac{1}{2}\tau_{xi}}\big)^2 + \big(\frac{y-b}{\frac{1}{2}\tau_{yi}}\big)^2 + \big(\frac{z-c}{\frac{1}{2}\tau_{zi}}\big)^2 + 1\Big]^{-1} \cdot \mathrm{i}\ , \end{aligned} \tag{11}$$

where M, N are the magnitudes of real and imaginary near fields, respectively. τ is the full width of half maximum (FWHM) of Lorentzian distribution for the x, y, and z components. a, b, and c refer to the displacement of Lorentzian function with respect to the origin, *i.e.*, the center of the near field. The imaginary part is nonzero in resonant Raman, otherwise it is zero. In our simulations, the FWHMs for x and y components are treated equally, and the FWHMs of the real and imaginary fields along the same axis are identical. The total near field distribution in Supplementary Equation 7 is obtained by adding the unit-magnitude external field to the diagonal elements to the local field tensor.

It is worth mentioning that the 3D Lorentzian distribution is normalizable in a finite space. In practice, a grid box that is significantly larger than the molecule's size is used for the calculation of the polarizability densities. The modeled near field is bounded within this box, which corresponds to a finite energy of the plasmon. The Raman polarizability densities decay exponentially away from a molecule and near the boundaries the polarizability densities are zero. The local integration of Raman polarizability densities (zz component

only) in the finite space is written as:

$$\begin{aligned}
&\int_{-r_{\max}}^{r_{\max}} \left[1 + F_z^{\mathrm{loc},z}(\mathbf{r} - \mathbf{R})\right] \cdot \delta\rho_{zz}^{(\alpha)}(\mathbf{r}) \cdot \left[1 + F_z^{\mathrm{loc},z}(\mathbf{r} - \mathbf{R})\right] d\mathbf{r} \\
&\approx \sum_{i}^{N} \left[1 + F_z^{\mathrm{loc},z}(\mathbf{r}_i - \mathbf{R})\right] \cdot \delta\rho_{zz}^{(\alpha)}(\mathbf{r}_i) \cdot \left[1 + F_z^{\mathrm{loc},z}(\mathbf{r}_i - \mathbf{R})\right] \cdot \Delta V \ .
\end{aligned} \tag{12}$$

Here $r_{\max}$ represents the boundaries of the box, N is the total number of grid points within the box, and ΔV is volume factor (volume of a unit grid cell). The locally enhanced Raman polarizability at a point ($\mathbf{R}$) is obtained by summing over all grids weighted by the volume element.

Supplementary Figures

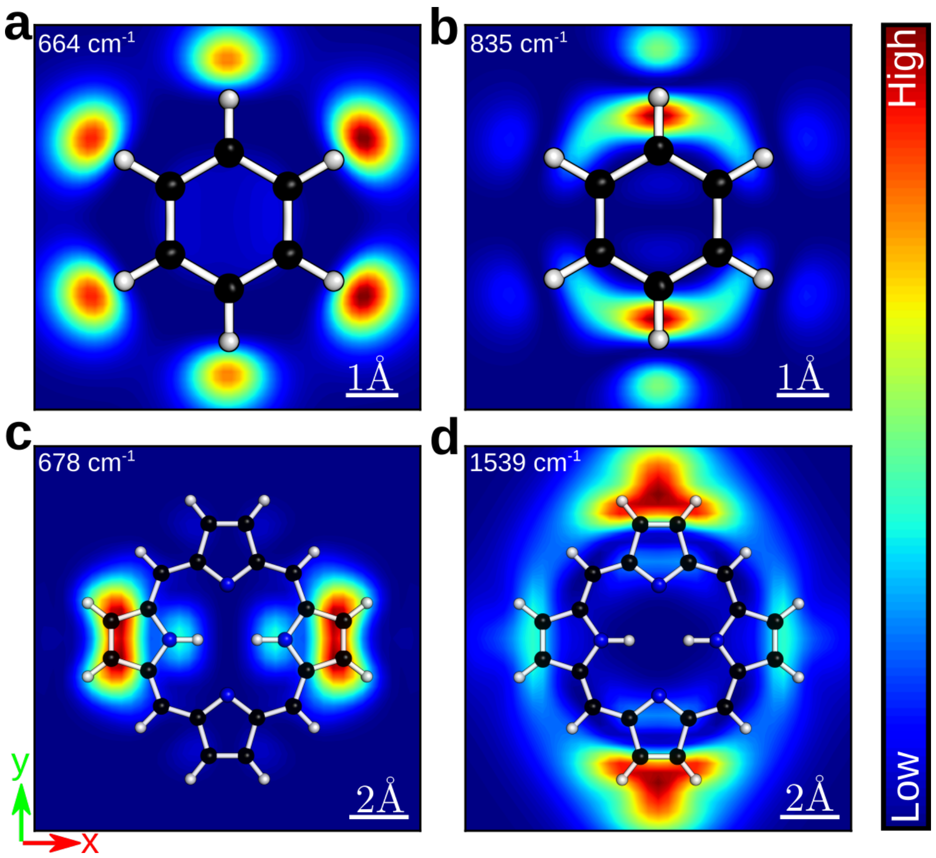

Supplementary Fig. 1: TERS images calculated using both alpha and $\mathcal{A}$-tensor densities in the same normal modes of benzene and prophyrin molecules discussed in the main text: (a,b) benzene, FWHM of field is 2.5 Å and 0.8 Å above the molecule plane; (c,d) porphyrin, FWHM of field is 3 Å and 1 Å above the molecule plane. The frequency of each normal mode is given on the left top of individual TERS image.

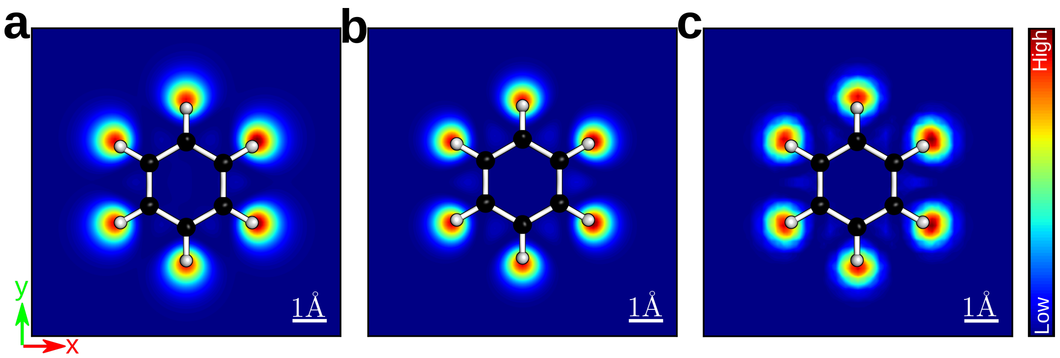

Supplementary Fig. 2: Comparison of benzene TERS images at 664 cm^{-1} obtained from the different near-field distributions. The near fields are in (a) 3D Lorentzianwith the FWHM of 1.3 Å, (b) 3D Gaussian with the FWHM of 1.3 Å, and (c) spherical step-function distributions with the diameter of 1.8 Å, respectively. The near fields are centered 1.0 Å above molecular plane

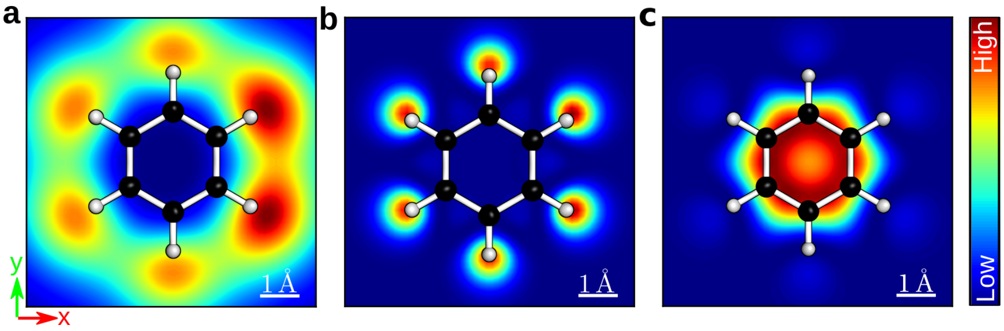

Supplementary Fig. 3: Comparison of TERS images of 664 cm^{-1} mode from different local integration volume. The center of integration volume above (a,b) 1 Å and (c) 2 Å away from the benzene plane, respectively. The FWHMs of 3D Lorentzian distributions on the xy-plane are (a) 5 Å and (b, c) 1.3 Å. The FWHMs of the z component are (a,c) 1.3 Å and (b) 5 Å.

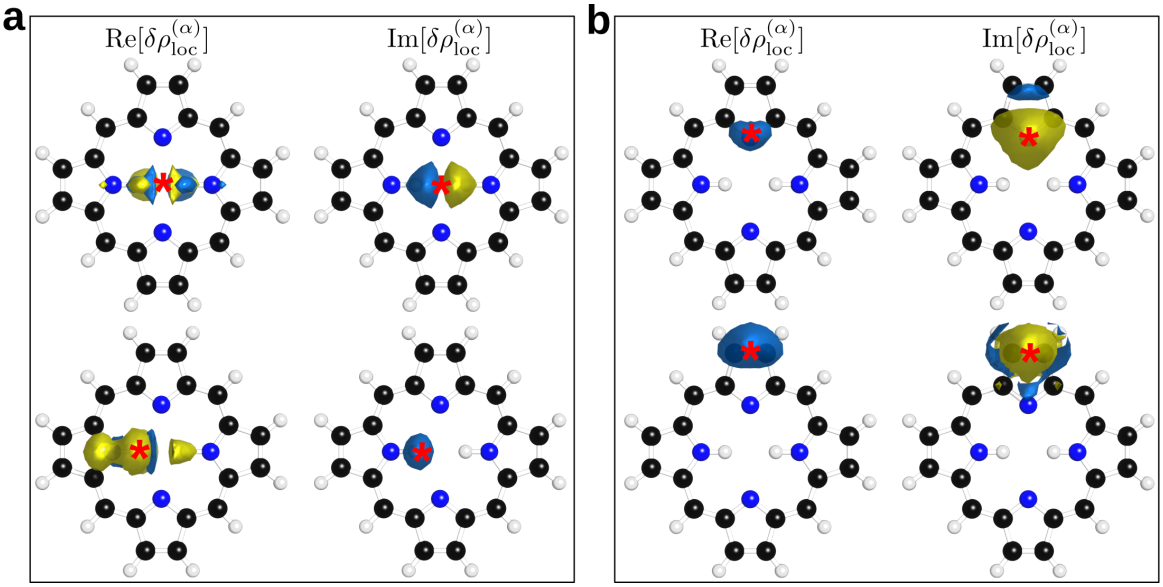

Supplementary Fig. 4: Locally enhanced Raman polarizability densities of modes (a) at 678 cm^{-1} and (b) at 1539 cm^{-1}. The confined near fields formulated in 3D Lorentzian distributions with the FWHMs of 2 Å for x, y, and z components are marked by red asterisks. The normalized real and imaginary densities are illustrated on the left and right columns in each panel, respectively. The isovalue is set to 0.2.

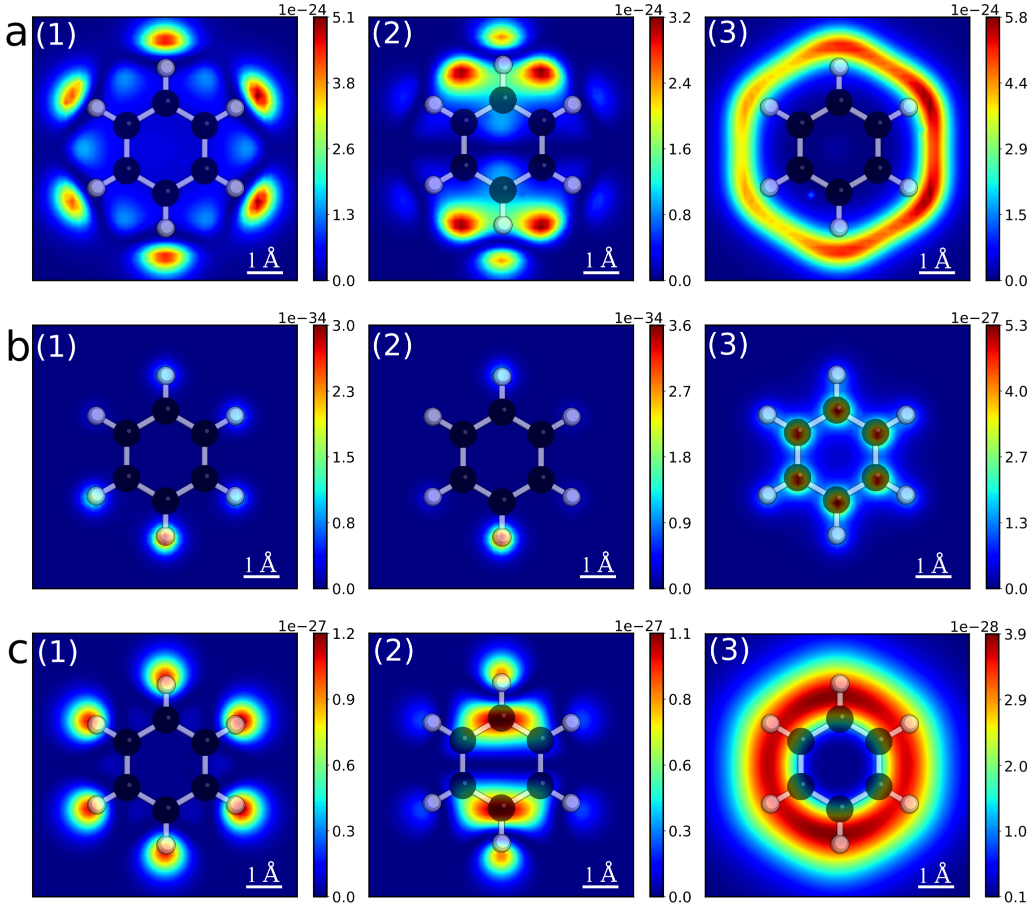

Supplementary Fig. 5: TERS images of benzene generated from three methods. Row a-c: DIM/QM, dressed Raman tensors, and LIRPD. Column 1-3: 664 cm^{-1} (symmetric out-of-plane bending), 835 cm^{-1} (anti-symmetric bending), and 988 cm^{-1} (in-plane ring breathing). The colorscale bars represent the calculated Raman scattering cross-sections.

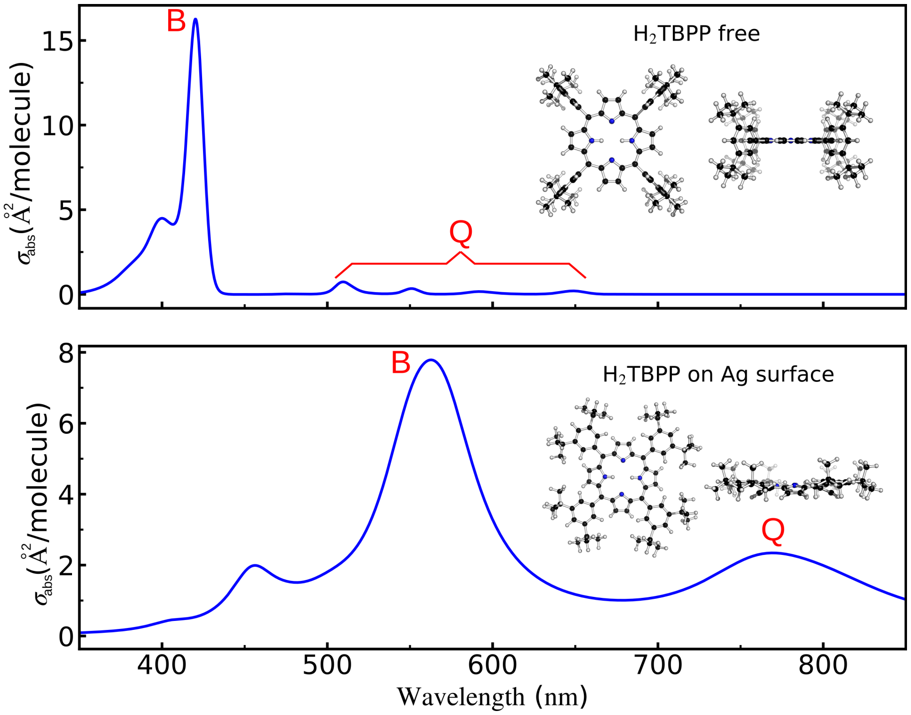

Supplementary Fig. 6: Comparison of absorption spectra of H_2TBPP in free state and adsorbed on Ag surface. The insert illustrates the structure of free H_2TBPP from the top and the side views (top panel), and the adsorbed structure is inserted as well (bottom panel).

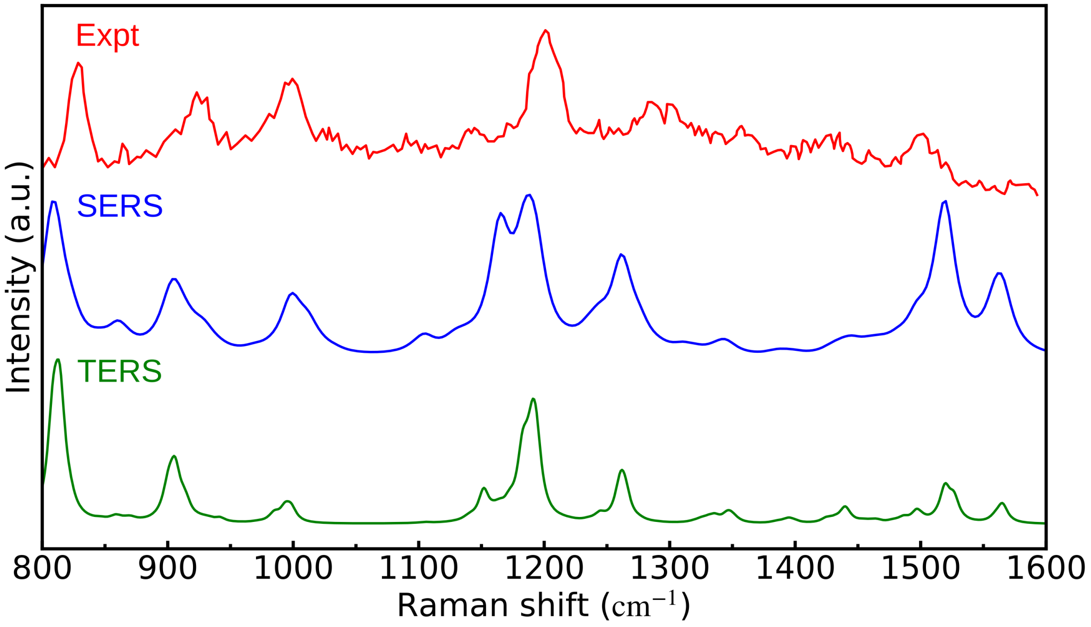

Supplementary Fig. 7: Comparison of resonant Raman spectra from the experimental measurements and theoretical simulations. The experimental TERS spectrum measured by the tip on a lobe in red. The simulated SERS spectrum obtained from the zz component of Raman polarizability in blue. The simulated TERS spectrum obtained from the LIRPD by using the integration volume in 3D Lorentzian distribution with $\Gamma_{x/y}$ = 12 Å and Γ_z= 6 Å in green. The experimental spectrum is adapted from ref. 11.

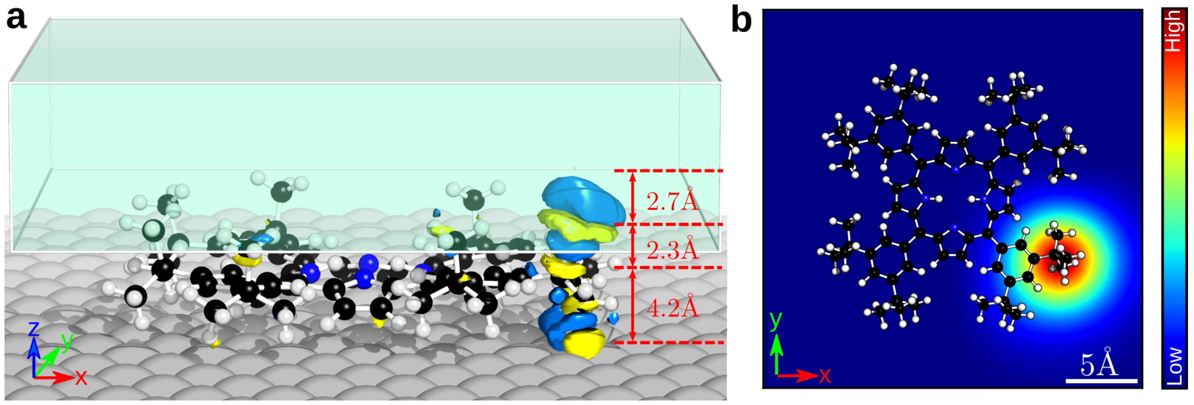

Supplementary Fig. 8: Resonant Raman polarizability densities and resonant TERS image of the mode at 1182.7 cm^{-1} (a) The Raman polarizability densities in real part are distributed throughout the scanning volume in green box with the thickness of 6 Å above the porphyrin plane in the perspective view. The distances among the Ag surface, the porphyrin plane, the top of molecule, and the center of integration volume are displayed in red. The densities are normalized and the absolute isovalue is set to 0.05 with the positive sign in blue and the negative sign in yellow. (b) The simulated resonant TERS images.

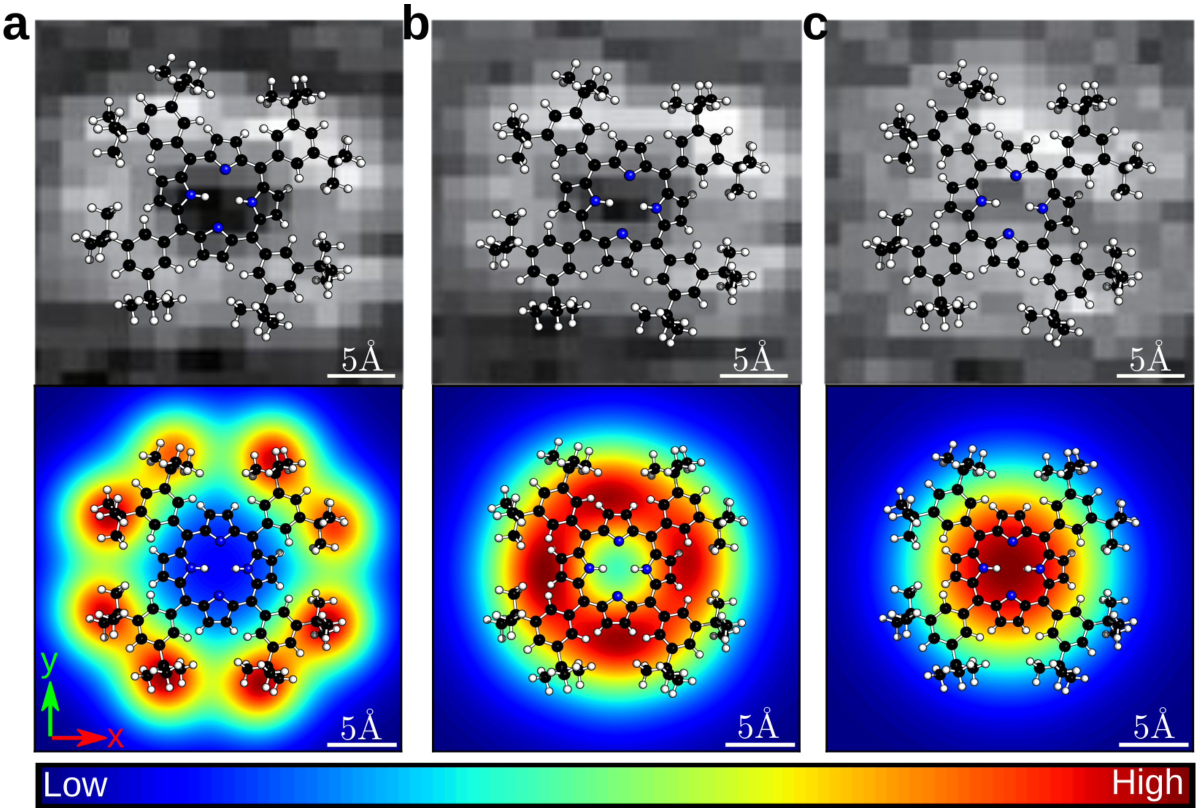

Supplementary Fig. 9: Comparison of resonant TERS image from the experimental measurements (top panel) and the theoretical simulations (bottom panel). The excitation energy at the B_y transition. The resonant TERS images are the combination of the modes at the frequencies around (a) 900, (b)990, and (c) 1520 cm^{-1}, respectively. The experimental images are adapted from ref. 11.

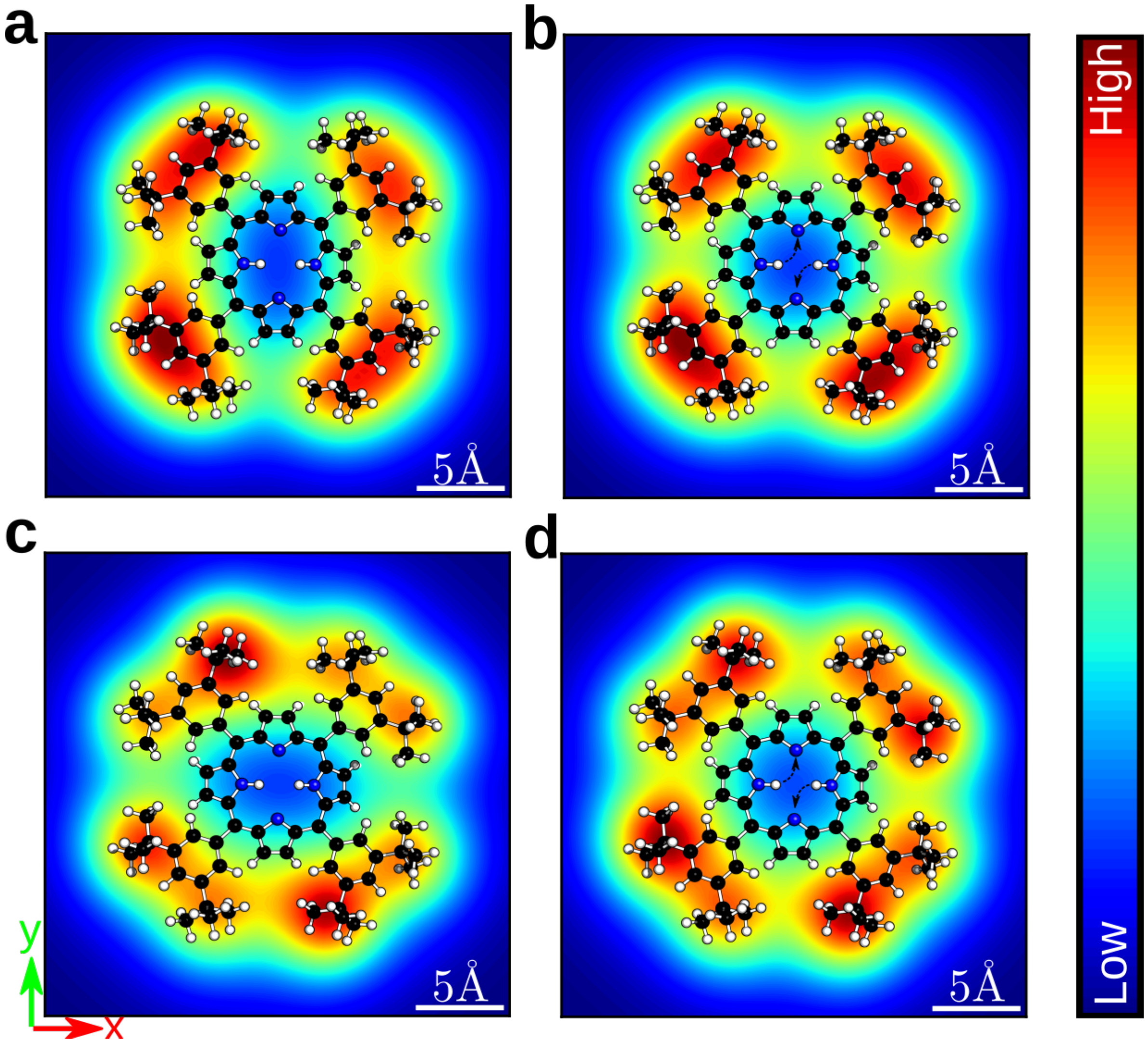

Supplementary Fig. 10: Comparison of resonant TERS image with and without tautomer contribution. The excitation energy at the B_y transition. The resonant TERS images are the combination of the modes at the frequencies around (a,b) 810 and (c,d) 1185 cm^{-1} (a,c) without and (b,d) with the tautomer contributions. The dashed arrow indicates the hydrogen transfer in tautomerization.

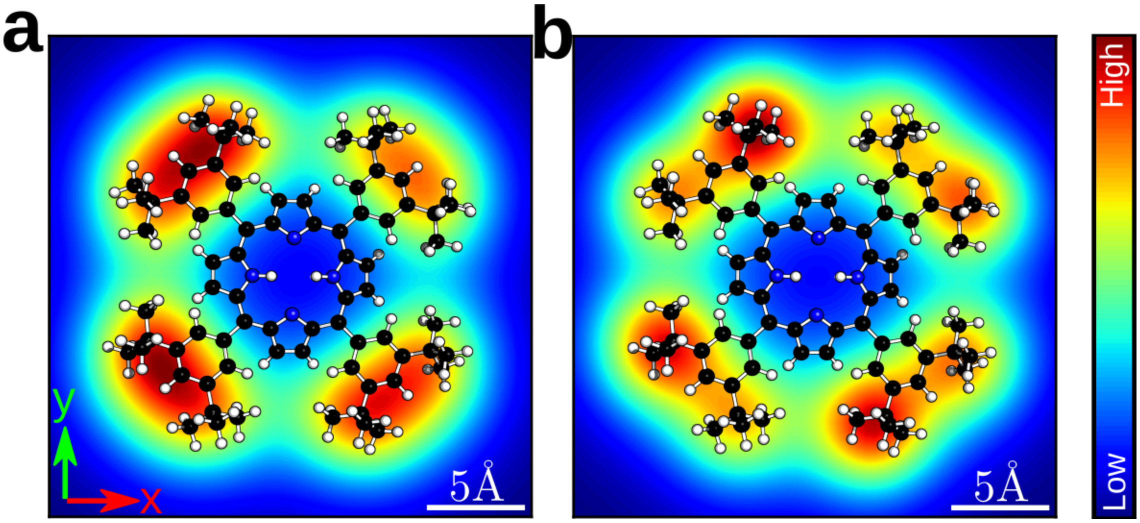

Supplementary Fig. 11: The resonant TERS images of H_2TBPP molecule with $Q_y(0,0)$ transition excited: (a) 810 cm^{-1}; (b) 1185 cm^{-1}. Each of the TERS images is averaged from the combination of prominent degenerate modes within a 20 cm^{-1} window, which are the same as in Figure 4 of the main text.

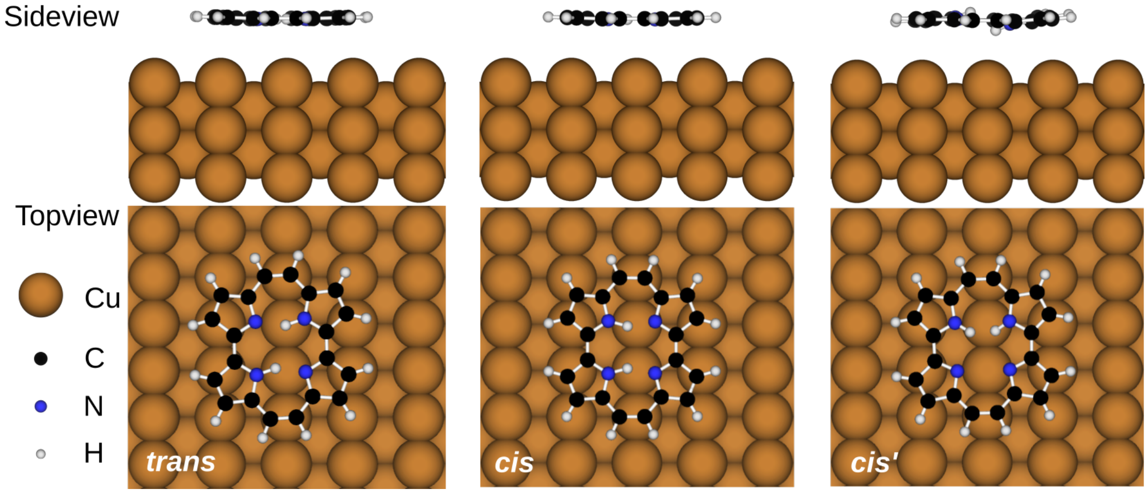

Supplementary Fig. 12: The sideview (top row) and topview (bottom row) of the optimized structures of the *trans*, *cis*, and *cis*′ configurations on a Cu(111) surface from left to right.

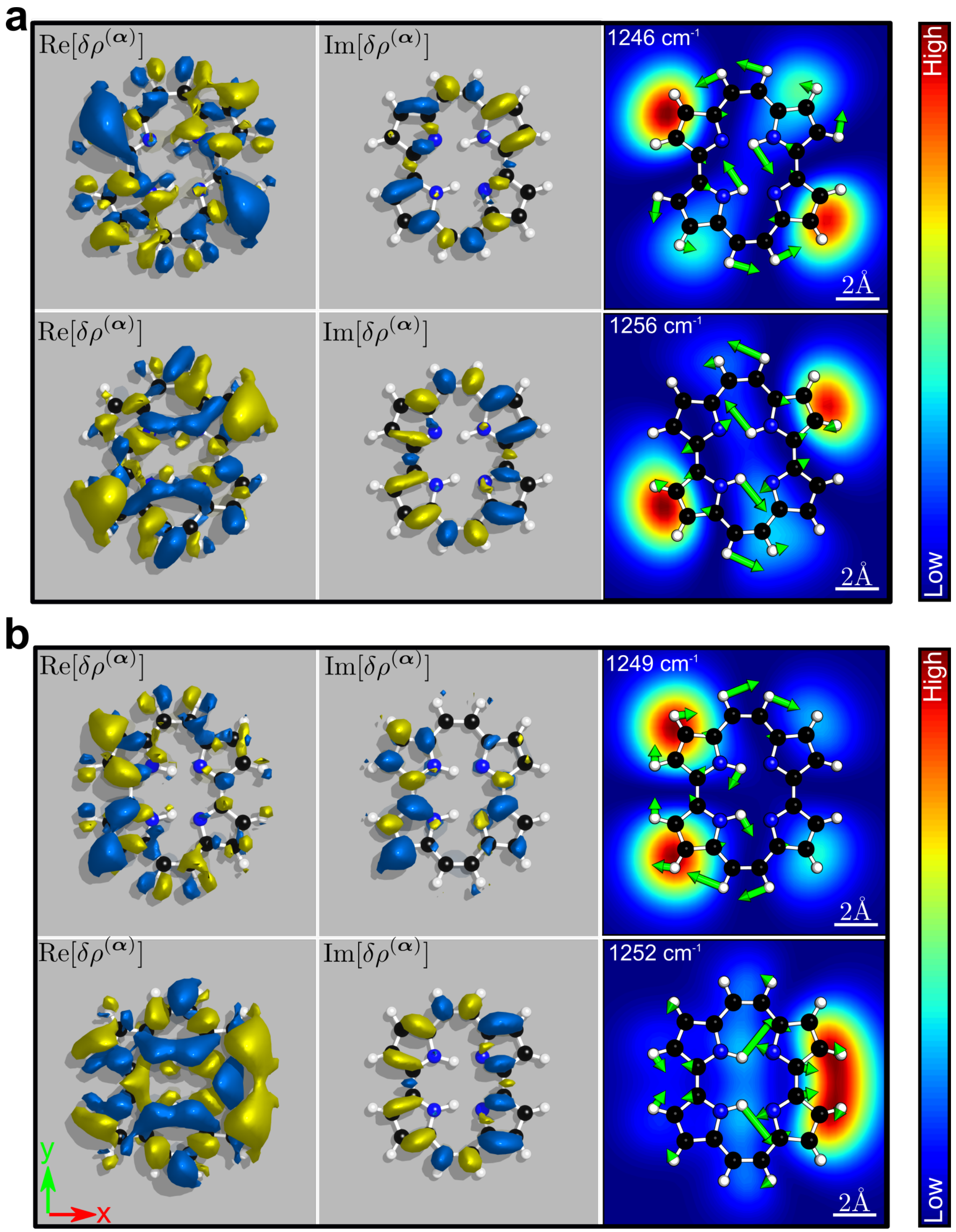

Supplementary Fig. 13: Raman polarizability densities and resonant TERS images of (a) *trans* and (b) *cis* porphycene molecules. The normalized real and imaginary density distributions with isovalue of 0.2 are on the left and middle columns in each panel, respectively. The vibrational frequencies and the corresponding TERS images are on the right columns.

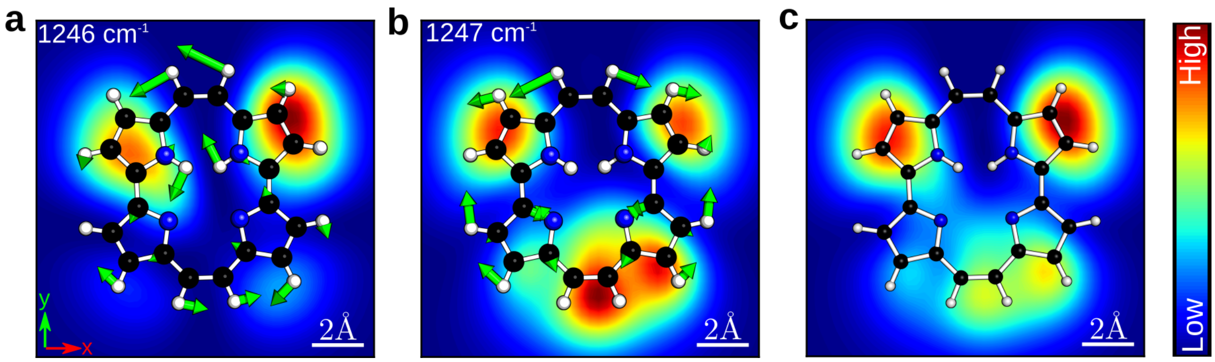

Supplementary Fig. 14: The resonant TERS images of the *cis*′ configuration of porphycene. TERS images of the modes at (a) 1246 and (b) 1247 cm^{-1}, and (c) the total image.

Supplementary Tables

Supplementary Table 1: The lowest five excited states and the properties including vertical transition energy (E), oscillator strength (f), and transition dipole moment (μ) in a.u.

Vertical transition	E (eV)	E (nm)	f	μ_x	μ_y	μ_z
1 $Q_x(0,0)$	1.54	802.3	0.218	-2.39	0.20	-0.44×10^{-3}
2 $Q_y(0,0)$	1.62	763.2	0.455	-0.26	-3.37	-0.42×10^{-2}
3	2.17	571.0	0.059	-1.05	-0.73×10^{-1}	-0.24×10^{-1}
4 $B_x(0,0)$	2.18	569.8	1.351	5.03	0.18	-0.70×10^{-2}
5 $B_y(0,0)$	2.24	551.4	1.056	-0.29	4.37	-0.19×10^{-2}

Supplementary Table 2: Fitting parameters of near-field distribution having the maximal magnitude along gap direction. The tips are in the shape of icosahedron and tetrahedron. All the parameters in Å.

	Icosahedron-to-substrate			Tetrahedron-to-substrate		
d_{gap}	d_{center}	$\Gamma_{x/y}$	Γ_z	d_{center}	$\Gamma_{x/y}$	Γ_z
4	2.0	8.5	2.5	1.9	6.7	2.8
5	2.6	10.1	2.9	2.5	7.8	3.0
6	3.3	12.1	3.3	3.2	8.9	3.4
7	4.0	13.6	3.9	4.0	9.8	4.0
8	4.9	14.9	4.6	5.0	10.2	4.6
9	6.0	15.7	5.5	6.0	10.4	5.3
10	7.0	16.1	6.4	7.1	10.4	5.9

Supplementary Notes

Supplementary Note 1 It is found that the hotspot pattern in TERS images is not sensitive to the exact shape of the near-field distribution. In Supplementary Figure 2, we show the TERS images of benzene at 664 cm^{-1} obtained from three different local field distributions: 3D Lorentzian, 3D Gaussian, and spherical step function distributions. The three TERS images are overall similar. The Lorentzian field leads to slightly bigger hotspots than the Gaussian, which is expected due to its fatter tail. The subtle roughness in the TERS image obtained by the step function is attributed to the discontinuity of the distribution.

Supplementary Note 2 In Supplementary Figure 1, we collectively compare the TERS images simulated by considering both alpha and $\mathcal{A}$-tensor densities (Supplementary Equation 7) with those generated from only alpha densities (in the main text). The most significant change by $\mathcal{A}$-tensor densities is seen in the benzene TERS images. The molecule itself is known for field-gradient evoked Raman scattering,[3] and the field band width required to reproduce DIM/QM images is 1.3 Å, below the scale of an atom. With $\mathcal{A}$-tensor densities included, the hotspots are moved slightly further away from the vibrating atom's positions (Supplementary Figure 1a vs. Main text Figure 2c). Similar behavior of the $\mathcal{A}$-tensor densities are also observed for porphyrin (Supplementary Figure 1b), although the hotspots are not moved as much as in the benzene case. It should be noted that for both benzene and porphyrin, the band width of the Lorentzian field is increased to reproduce DIM/QM images when $\mathcal{A}$-tensor densities are considered. This is expected, especially for atomically confined field, because the representation of the true near field becomes increasingly accurate when the field gradient (or even higher-order terms) is included (Supplementary Equation 7). However, the effect of $\mathcal{A}$-tensor densities becomes trivial when the field confinement is beyond the atomic scale.

In short, we have shown that including the $\mathcal{A}$-tensor densities does not drastically change the overall hotspot patterns in TERS images. The $\mathcal{A}$-tensor densities tend to drive the

hotspot away from the vibrating atoms, especially for atomically confined near field and field-gradient active modes. But for less confined near field, $\mathcal{A}$-tensor densities contribute is trivial. Therefore, we conclude using alpha densities alone in the LIRPD approach is sufficient to model TERS imaging for most cases.

Supplementary Note 3 TERS image is strongly dependent on the integration volume in terms of size and position. In simulations, the height from the field center to molecular plane is fixed. Supplementary Figure 3 illustrates the correlation between TERS images and integration volumes. The benzene molecule lies down on a silver surface. With the FWHM increasing from 1.3 to 5.0 Å on xy-plane, the image is blurred (Supplementary Figure 3a). Keeping FWHM at 1.3 Å on the xy-plane and increasing FWHM to 5 Å for the z component do not blur the TERS image (Supplementary Figure 3b), which means the image resolution is more sensitive to the field distributed on scanning plane. It is also shown that the drastic changes take place as the integration volume moves slightly up with respect to the molecular plane in Supplementary Figure 3c, which means the scanning height is of importance for TERS imaging as well. These findings suggest that distributing near fields within atomic dimensions over an appropriate imaging plane is the key to the atomic resolution in TERS images.

Supplementary Note 4 To further demonstrate the quality of the approximation made in LIRPD method, we compare it with the method of reported in the original TERS imaging paper[11] and later adopted in the studies of chemistry in nanocavities.[12,13] The gist of that method is to apply the localized near field to the Raman tensor. The idea can also be related to the dressed tensors formalism. The main difference is that the near field tensor dresses the free-molecular polarizability derivatives w.r.t. atomic displacements in the given normal mode, rather than w.r.t. the normal mode coordinate (Q_k). Also, no multipole expansion is used. We refer to that method as dressed Raman tensor.

In Supplementary Figure 5, we show the calculated TERS images of the same three benzene modes as reported in ref. 4, namely, symmetric bending, anti-symmetric bending, and ring-breathing. The characteristics of these three TERS images are 1) the hotspots are slightly away from the atoms, and 2) normal Raman inactive modes are activated by the strong near field gradient.

Comparing with the DIM/QM method (row a), we find the dressed Raman tensor method (row b) results in the hotspots highly localized on atoms instead of being slightly away. This is expected because the polarizability derivatives and the near field enhancement are evaluated at each atom's position. Moreover, the dressed Raman tensors method does not correctly describe normal Raman inactive modes. The cross-sections of the 664 cm^{-1} and 835 cm^{-1} modes in the dressed Raman tensors method (Supplementary Figure 5 b1 and b2) are merely zero compared with the Raman active mode at 988 cm^{-1} (b3), and the patterns in the TERS images are trivial. In short, the dressed Raman tensors method does not appear to be able to accurately describe the local perturbation of the molecule due to the confined near field in TERS.

However, using the same field distribution as in the above dressed Raman tensors method, we find the LIRPD method proposed in this work qualitatively reproduces the key characteristics of the benzene TERS images. The three TERS images all have comparable cross-section maxima. The hotspot positions are already off the atoms with only the alpha densities included. Moreover, include the $\mathcal{A}$-tensor densities in LIRPD further moves the hotspots away from the atoms, which was discussed in the previous section. Therefore, we conclude that the LIRPD model is a decent approximation to TERS selection rules, and helps explain the mechanism underlying the TERS images.

Supplementary Note 5 The integration cancellation happens when the neighboring densities hold opposite signs. For the mode at 678 cm^{-1}, we see the symmetric enhanced density distribution when the tip is above the center of porphyrin. The integrated densities will be

zero leading to a silent Raman signal (Supplementary Figure 4a top). When the tip is off the porphyrin center, we see the symmetric distribution is broken, which leads to a strong Raman intensity (Supplementary Figure 4a bottom). For the mode at 1539 cm^{-1}, two locally enhanced density distributions were selected to interpret their relative Raman intensities. We found the maximal absolute value of the locally enhanced densities is 4 times larger on the C-C bond (Supplementary Figure 4b bottom) than on the nitrogen atom of the pyrrole moiety (Supplementary Figure 4b top). It is the reason why the hot spots are distributed on the C-C bond of the pyrrole moieties rather than on the nitrogen atom.

Supplementary Note 6 According to the previous study,[5] H_2TBPP preferentially adopts concave configuration when adsorbed on the Ag(111) surface under the experimental conditions. We therefore performed the calculations based on the reported concave configuration.

The simulated absorption spectra of H_2TBPP in free state and adsorbed on a Ag(111) surface are plotted in Supplementary Figure 6. The Q-band absorption of free H_2TBPP spans from 500 to 660 nm, and the B-band absorption is centered at 420 nm. In the adsorbed state, both Q and B bands are red shifted by more than 100 nm. It shows the broad Q-band is centered around 770 nm with relatively strong absorbance cross section (Å^2/molecule) even though the Herzberg-Teller term is absent. It is because the high symmetry (D_{4h}) in free state is broken due to the strong interaction between molecule and substrate in the adsorbed state. The planar porphyrin fragment is distorted due to the steric hindrance from the phenyl moiety substitutes which parallel orientate on Ag surface. The B-band at 562 nm is close to the incident light at 532 nm in experiment.[11] As show in Supplementary Table 1, the B_y(0,0) band excitation at 551.4 is more likely to be excited under the experimental conditions than the previously assumed Q_y(0,0) band.

To gain insight into the near-field distributions in the plasmonic junctions, we revisit the correlation between plasmonic gap and near field in the tip-to-substrate junction.[14] We collected the fitting parameters of near field in terms of the position (d_{center}) where the field

magnitude is a maximum and the FWHM of near-field distributions of three components (Γ) at d_{center} correlated with the gap distance (d_{gap}) at the incident field polarized along z-axis in Supplementary Table 2. With the increasing gap distance, the field center becomes closer to the tip and the near-field bandwidths become broader. The bandwidths of the x and y components are equivalent and much broader than that of the z component. The ratio of bandwidths between x (y) and z components is generally smaller than 1/2 for the tips with different curvatures. Additionally, the previous study has shown that the near field is further squeezed and pushed toward tip in the presence of a molecule in the junction due to the screening effects.[15] Considering the near-field simulation results and orientation of H_2TBPP adsorbed on Ag substrate (see Supplementary Figure 7), the integration volume in the shape of disk with $\Gamma_{x/y}$= 12 Å and Γ_z= 6 Å is centered at 9.2 Å above Ag surface.

To illustrate how the resonant Raman polarizability densities spatially distributed throughout the scanning volume which is the entire space for the integration via scanning, we take one normal mode at 1182.7 cm^{-1} for example. The center of scanning space is located 2.7 Å above the top of H_2TBPP. A portion of the resonant Raman densities localized on the top of a butyl group are taken into account in the local integration, as shown in Supplementary Figure 7a. As a result, the TERS image arising from the Raman spectral mapping on the midplane of scanning volume is featured by a hotspot on a butyl group (Supplementary Figure 7b).

The simulated SERS and TERS spectra incorporated with experimental TERS spectrum measured on a lobe are demonstrated in Supplementary Figure 8. The SERS spectrum obtained from the zz component of Raman polarizability shows most of important modes. However, the intensity at 1520 cm^{-1} is quite strong compared with other modes. The TERS simulated by the LIRPD method is in good agreement with the experimental spectrum. All the important peaks and relative intensities are well reproduced. It confirms the LIRPD is a reliable approach for simulating both TERS image and spectrum.

The simulated TERS images at 900, 990, 1520 cm^{-1} are consistent with the experi-

mental TERS mapping results. The characteristic bright four-lobe pattern is hold in low-wavenumber modes. More importantly, we see the central dark area gets smaller from low to high wavenumbers and eventually collapses to a hotspot in the center, which agrees the experimental results. In addition, it is speculated that the high-wavenumber modes above 1210 cm^{-1} contain more contributions from the porphyrin core, but the experimental TERS mapping were insufficiently resolved to conclusively make this conclusion.[11] Our simulated TESR image at the frequency of 1520 cm^{-1} clearly shows the hotspot arising from porphyrin core. It confirms again the LIRPD approach and the assumed near field used for local integration are appropriate.

We now explore the effect of H_2TBPP tautomers on its TERS images. The TERS images with hydrogen tautomerization are given in Supplementary Figure 10b and 10d at frequencies at 810 and 1185 cm^{-1}. In comparison, the TERS images of one specific configuration are provided in Supplementary Figure 10a and 10c. We find that averaging the degenerate modes of only one configuration is already sufficient to match the experimental images, and the enforced tautomerization of the porphyrin leads to nothing more than slightly more symmetric patterns.

We also examined the TERS images under the Q_y(0,0) excitation as was proposed in the experiment (Supplementary Figure 11). However, no significant difference is identified in comparing the TERS images under B_y(0,0) and the Q_y(0,0) excitations. This is expected because the transition dipoles of both these two excited states are associated with the base porphyrin ring, but the TERS tip mainly probes the butyl groups and is not likely to reach the bottom of the molecule. Moreover, the transition dipole moments are mostly in the xy plane with very small z contributions (Supplementary Table 1), whereas in TERS the z scattering is the dominant component detected. As a result, the effects of these two resonance states are equally small. Therefore, we conclude that the TERS images of H_2TBPP is insensitive to the resonant Raman effect unless the butyl groups are excited.

Supplementary Note 7 In Supplementary Figure 13, the left and middle columns illustrate the topview of Raman polarizability densities in scanning volumes. Correlating density distributions with TERS images, we see the hotspot distributions in the images are similar to the real density distribution patterns. Comparing individual TERS images of *cis* with its counterparts in *trans*, we see the subtle differences in the tautomer structures are well identified.

The TERS image of the band at 1250 cm^{-1} and individual mode contributions for the cis' configuration are illustrated in Supplementary Figure 14. The total image is a combination of two modes. In the cis' configuration, the prominent density distributions are related to the *ortho*-hydrogen vibrations. The mode with the large displacements of the central hydrogen atoms provides the major contribution to the total TERS image, where the hot spots are distributed on the adjacent pyrrole moieties of the cis' porphycene. It is evident that the image pattern follows the configuration of the two central hydrogens which bind to the *ortho*-pyrrole in the cis' porphycene.

Supplementary References

1. Gross, E. K. U., Dobson, J. F. & Petersilka, M. *Density functional theory of time-dependent phenomena*, 81–172 (Springer Berlin Heidelberg, Berlin, Heidelberg, 1996).

2. Payton, J. L., Morton, S. M., Moore, J. E. & Jensen, L. A hybrid atomistic electrodynamics–quantum mechanical approach for simulating surface-enhanced raman scattering. *Acc. Chem. Res.* **47**, 88–99 (2014).

3. Chulhai, D. V. & Jensen, L. Determining molecular orientation with surface-enhanced raman scattering using inhomogenous electric fields. *J. Phys. Chem. C* **117**, 19622–19631 (2013).

4. Liu, P., Chulhai, D. V. & Jensen, L. Single-molecule imaging using atomistic near-field tip-enhanced raman spectroscopy. *ACS Nano* **11**, 5094–5102 (2017).

5. Duan, S. *et al.* Theoretical modeling of plasmon-enhanced raman images of a single molecule with subnanometer resolution. *J. Am. Chem. Soc.* **137**, 9515–9518 (2015).

6. Duan, S., Tian, G. & Luo, Y. Visualization of vibrational modes in real space by tip-enhanced non-resonant raman spectroscopy. *Angew. Chem., Int. Edit.* **55**, 1041–1045 (2016).

7. Barron, L. D. *Molecular Light Scattering and Optical Activity* (Cambridge University Press, 2004), 2nd edn.

8. Janesko, B. G. & Scuseria, G. E. Surface enhanced raman optical activity of molecules on orientationally averaged substrates: Theory of electromagnetic effects. *J. Chem. Phys.* **125**, 124704 (2006).

9. Chulhai, D. V., Hu, Z., Moore, J. E., Chen, X. & Jensen, L. Theory of linear and nonlinear surface-enhanced vibrational spectroscopies. *Ann. Rev. Phys. Chem.* **67**, 541–564 (2016).

10. Hu, Z., Chulhai, D. V. & Jensen, L. Simulating surface-enhanced hyper-raman scattering using atomistic electrodynamics-quantum mechanical models. *J. Chem. Theor. Comput.* **12**, 5968–5978 (2016).

11. Zhang, R. *et al.* Chemical mapping of a single molecule by plasmon-enhanced raman scattering. *Nature* **498**, 82–86 (2013).

12. Benz, F. *et al.* Single-molecule optomechanics in “picocavities”. *Science* **354**, 726–729 (2016).

13. Shin, H.-H. *et al.* Frequency-Domain Proof of the Existence of Atomic-Scale SERS Hot-Spots. *Nano Lett.* **18**, 262–271 (2018).

14. Chen, X. & Jensen, L. Morphology dependent near-field response in atomistic plasmonic nanocavities. *Nanoscale* **10**, 11410–11417 (2018).

15. Lee, J. *et al.* Tip-enhanced raman spectromicroscopy of co(ii)-tetraphenylporphyrin on au(111): Toward the chemists' microscope. *ACS Nano* **11**, 11466–11474 (2017).
